# Looking, pointing, and talking together: How dyads of differential expertise coordinate attention during conversation

**DOI:** 10.1371/journal.pone.0315728

**Published:** 2024-12-19

**Authors:** Lucas Haraped, Stefan E. Huber, Walter F. Bischof, Alan Kingstone

**Affiliations:** 1 Department of Psychology, University of Innsbruck, Innsbruck, Austria; 2 Department of Psychology, University of Graz, Graz, Austria; 3 Department of Psychology, University of British, Columbia, BC, Canada; University of Giessen: Justus-Liebig-Universitat Giessen, GERMANY

## Abstract

When people discuss something that they can both see, their attention becomes increasingly coupled. Previous studies have found that this coupling is temporally asymmetric (e.g., one person leads and one follows) when dyads are assigned conversational roles (e.g., speaker and listener). And while such studies have focused on the coupling of gaze, there is also evidence that people use their hands to coordinate attention. The present study uses a visual task to expand on this past work in two respects. First, rather than assigning conversational roles, participants’ background knowledge was manipulated (e.g., expert and novice) to elicit differential roles inherent to the conversation. Second, participants were permitted to gesture freely while interacting. Cross Recurrence Quantification Analysis with data from mobile eye trackers and manually coded pointing gestures revealed that although more knowledgeable participants dominated the dialogue by talking and pointing more, the symmetry of coupled behaviors (gaze and pointing) between participants remained fixed. Asymmetric attentional coupling emerged, although this was dependent on conversational turn taking. Specifically, regardless of background knowledge, the currently speaking participant led attention, both with the eyes and with the hands. These findings suggest stable, turn-dependent interpersonal coupling dynamics, and highlight the role of pointing gestures and conversational turn-taking in multimodal attention coordination.

## 1. Introduction

As social beings, humans are able to coordinate their attention and actions remarkably well among one another to achieve common goals [1]. In natural dialogue, such coordination is most often achieved through the simultaneous use of several different communicative channels such as gestures [2–4], eye gaze, and conversational turn taking [5–7]. During social interactions, people regularly and mutually influence each other within and across channels. The result is that people’s communicative behaviors, as well as their attention, become coupled over time and exhibit properties of a self-organized, complex dynamical system [8]. In this paper, we use the term ‘coupling’ to broadly refer to the phenomenon of two (or more) behaviors dynamically and reciprocally influencing one another (but see [9] on the ambiguity of definitions in related concepts).

Interpersonal coordination through coupled behaviors paves the way for efficient communication. Within a set of behavioral states that a joint system between co-actors can assume over time, coupling behaviors leads to a reduction in the behavioral system’s overall degrees of freedom, making interlocutors’ predictions about each other easier [10, 11]. The resulting coordinative structure between individuals therefore eases the burden on each individual cognitive system and embodies the goals of their now joint action system [12]. As long as communication is interactive [13], this essentially allows people to self-organize [14] and synergistically perceive, act and think together as an efficient unit rather than as a set of individual, connected parts [8, 15].

The coupling of lexical, prosodic and speech/pause patterns in dialogue, for instance, predicts collective task performance [16]. In turn, when task constraints get more difficult, the coupling of some matched behaviors among interlocutors, such as emotional display, gestures and postural movement has been shown to increase, adding to the notion that behavioral coupling offers an adaptive capability for interacting minds [11, 15].

The above also appears true for the coupling of visual attention. When working on a joint task on a shared screen for instance, co-actors show higher gaze coupling with each successive round of the task they complete, which goes hand in hand with their performance in each round [17]. Similarly, when two interlocutors are given the same visual context on a shared screen while one of them is monologuing and the other one is listening, a subsequent comprehension test for the listeners shows that higher-scoring listeners’ eye movements are also more highly coupled to the speakers’ eye movements [18]. In turn, gaze coupling among participants engaged in dialogue proves to be stronger when interlocutors received matching compared to mismatched background knowledge prior to the conversation [19]. This underlines that attentional coupling reflects the alignment of two minds.

### 1.1 Asymmetries in behavioral coupling

An instance of symmetric coupling occurs when two peoples’ gaze towards something coincides without one co-actor leading the other. During dialogue for example, when two individuals are being shown the same visual context to talk about, their gaze is coupled most strongly at lag 0, indicating that most of the time conversing interlocutors look at the same thing at the same time [19].

In contrast, asymmetric coupling suggests a directionality in attentional coupling, with one leading and the other following in time [20]. Prior research has typically observed asymmetric coupling, with participants being assigned specific roles [e.g., 15, 17]. For instance, Richardson and Dale [18] found that listeners were most strongly coupled to the gaze of speakers by a lag of about 2 seconds. While the practice of assigning conversational roles can lead to well controlled and observable asymmetric conversational dynamics, the ecological validity of the approach may suffer from the fact that each co-actor is constrained by the respective affordances of their assigned role. This begs the question to what extent asymmetric attentional dynamics occur in natural conversation where asymmetry is not an inherent part of the task itself but tied to differences that each individual brings to the task.

### 1.2 Multimodal attentional coupling: Gaze and deictic gestures

While gaze behavior marks the current focus of an individual’s overt visual attention and signals it to others [21], deictic gestures (i.e., pointing) convey a more intentional and clear directional reference. Such gestures are used by human infants as young as 12 months [22] and though they are not observed for non-human primates in the wild [23] they have been reported for non-human primates who have been raised with humans and taught sign language [e.g., 24]. This suggests deictic gestures to be a key feature of human language development. Often occurring closely in time with a pointer’s gaze, these gestures disambiguate the current verbal and visual focus of attention by guiding the eyes of others [3, 4, 25–27]. They thus provide an efficient resource to structure interaction in time and space, as they signal to another individual both when and where to look during ongoing conversation [28]. The coupling dynamics of gestures and gaze is therefore suggestive of the coordinated stability of attention between individuals. Indeed, the notion that “hand-gaze” coordination (rather than “gaze-gaze” coordination) may serve as an alternate route to navigating joint attention underpins recent experimental studies. One such study found that people who observe and try to copy actions of a videotaped demonstrator solving a manual puzzle task were better at doing so when their attention was coupled with the hand actions of the demonstrator [29]. Another study showed that people do not always look at the object that they are about to point at, and that this incongruency hampers the ability of others to follow the pointing gesture with their attention [25]. Notably, these two studies used unidirectional (non-interactive) and trial-based designs.

To our knowledge, only two studies have investigated “hand-gaze” coordination as a possible alternative pathway to attentional coupling during ongoing interaction. Yu and Smith [30, 31] investigated infants and their caregivers manually interacting with objects. They found that during playful manipulation of toys, infants’ and caretakers’ hand- and eye-movements provided redundant information of attentional focus. This allowed them to coordinate attention without the need for words or directing gaze towards the other’s face.

### 1.3 Present study

The aim of the present investigation is to extend research on the symmetry of attentional coupling on two fronts. First by investigating the extent that differences between co-actors’ knowledge—rather than their predefined roles, as often done in previous studies—translates into asymmetries in attentional coupling. Second, by investigating attentional coupling as a multimodal phenomenon comprising gaze behavior, deictic gestures, and conversational turn-taking in a scenario where participants are physically co-present. To do so, we manipulated the task-relevant background knowledge of participants who jointly solved a visual sorting-task while being co-located, wearing mobile eye trackers, and permitted to gesture freely while conversing with one another. Crucially, rather than assigning fixed roles on how to act in a task, the manipulation of a tertiary variable—background knowledge—was used. The effectiveness of this manipulation has been demonstrated in the past in a non-verbal tapping task, where the goal was for two co-actors to synchronize their taps to specific targets [32]. With only one co-actor having knowledge about the correct location of these targets, arm movements between co-actors became synchronized, asymmetrically, with the taps of the less knowledgeable person lagging behind the more knowledgeable individual.

When it comes to attentional coordination, having matched background knowledge has been shown to increase symmetric gaze coupling in conversing interlocutors [19]. Hence, we speculate that having different levels of background knowledge might lead to asymmetric coupling of attention. This would be in line with the notion that recognition of leadership is bound to the knowledge an individual has in a particular valued domain [33–35]. In social interactions for example, people with high perceived or objective competence have more speaking time and are more expressive [36] and receive the greater share of attention [37].

In the current study, we aim to induce differential knowledge by assigning each participant from a dyad to different trainings before they complete their joint task. One training conveys knowledge relevant to the joint task (those participants are being called “trained”), while the other one conveys trivial knowledge only seemingly relevant to the task (those participants are being called “untrained”, see S1 Appendix for more information). This leads to four distinct hypotheses, which we outline below.

First, we hypothesize that **trained individuals speak more than untrained individuals (H1).** Second, that **trained individuals lead with their gaze in relation to their untrained counterpart’s gaze**, as their differential task-relevant background knowledge might lead to asymmetric gaze coupling **(H2)**.

Notably, there are two types of conversational roles subjects can be in during their interaction, the role of the trained or the untrained participant (for the whole conversation) and, at the same time, in the role of the current speaker or the current listener (at each given moment during the conversation). This introduces another level of analysis for the third hypothesis, which takes into account the temporal factor of conversational turn taking: As earlier studies showed symmetric gaze coupling in dialogue [19], yet asymmetric gaze coupling during monologue, where speakers’ gaze led listeners’ gaze [18], we thus further propose that asymmetries might be found in dialogues when taking conversational turns into account. More specifically, we hypothesize that **whoever speaks during a certain period of the conversation leads the dyad’s joint focus of attention during that time more than their listening counterpart (H3).** It is worth noting that H2 and H3 can stand independently, i.e., either, both, or neither can be supported. Finally, as in real world social interactions, deictic gestures are often expressed in close temporal proximity with one’s own and others congruent gaze [3, 25]; thus we investigate hand-gaze coupling [30] as a pathway to attentional coordination. We hypothesize, in keeping with H1, that **trained individuals point more than untrained individuals** (**H4).**

There are several outstanding unknowns that we also examine. First, how are one’s own gaze and pointing coordinated? Second, how does pointing as a form of attentional signaling impact the gaze of the other? A recent study reports that individuals who are pointing at an object during conversation look at that same object directly before pointing at it roughly 50% of the time [25]. Also, a study using a trial-based task investigated the intrapersonal timing of pointing and gaze onsets during unidirectional (not-interactive) direction signaling towards relevant targets. This revealed that in typically developed adults, the onset of gaze towards an intended target precedes the onset of a pointing gesture towards that same target on average by roughly 200 ms [26]. Adding to this, our approach should allow a more fine-grained analysis of the dynamics between gaze and pointing during an ongoing interaction. Finally, there is the question of what, if any, role training will play in this gaze-pointing relationship. For example, is the temporal coupling of gaze and pointing equivalent for trained and untrained individuals; and is their impact on the other equivalent or different?

## 2. Method

### 2.1 Participants

Sixty male, first-year undergraduate psychology students from the University of Innsbruck, Austria were recruited to participate in the 30-minute training course. We chose to include only male participants because participant gender has been shown to influence joint decisions [38] as well as gaze behavior during interactions [39]. 56 of the participants could be matched into 28 dyads of differential competence, as four of the participants withdrew from further participation. Ten dyads had to be discarded for the following three reasons that led to unusable eye-tracking data. In 6 instances participants self-reported non-normal vision, 3 dyads were lost due to technical failure with the eye trackers (failed calibration or failed recording), and in one instance one participant failed to follow instructions properly. This resulted in data from 18 dyads (36 participants) being used for data analysis, which is similar to studies using comparable methodological approaches [19]. The recruitment period for this study was October 7^th^ to October 18^th^ 2019. The study was approved by the ethics board of the University of Innsbruck, and students received course credit for participating and provided written informed consent prior to participation.

### 2.2 Task

To facilitate cooperative discussion, a ranking task similar to a survival task [40] was used, where participants are asked to rank a set number of items by their relative importance. The idea behind this task is that although there exists a theoretical correct order of items, there is still enough ambiguity to allow discussion. In the task used in this study, participants had to rank animals by their apparent age, as this required participants to jointly look at items of interest (i.e., pictures of those animals). While it is fairly straightforward to explain, teach and converse about visible age differences of the animals, few to no participants had domain specific knowledge beforehand. Specifically, participants had to rank 9 pictures of animals arranged in a 3 x 3 matrix, by judging features like color, size, and posture. Each item was labeled with a letter which participants were asked to jot down on a piece of paper in the order they agreed on.

We tested different types of stimuli and arrangements in pilot tests and chose this task for a number of reasons: The decision-making process here relies on visual information from pictures, which has been shown to provide enough ambiguity within and between each other to create the need to look at and compare the pictures throughout the whole task. Further, a living being’s age is a well-known concept where one can be wrong or right about when ranking it (different to topics where personal taste plays a role), yet it provides enough ambiguity due to its novelty to participants to allow for training effects and room for discussion. This made the topic ideal to manipulate levels of expertise.

### 2.3 Apparatus

**Fig 1:**
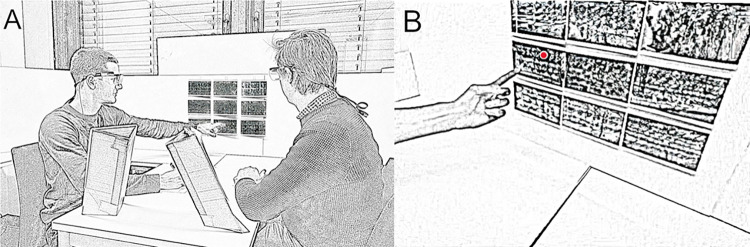
Depiction of experimental setup. (A) Schematic depiction of two participants (re-enacted), a trained and an untrained one, interacting by looking, pointing, and talking while referencing objects of interest to come to a joint decision. On the cardboard stands in the foreground, participants’ individual notes are placed close to eye-height to mitigate data loss. (B) Depiction of the right participant’s eye tracker video feed showing a red dot indicating the gaze position as well as the left participant’s pointing hand.

The task setup consisted of a 60 x 120 cm table with a swivel chair on each long side (see Fig 1). On one short side of the table, at a distance of approximately 60 centimeters and in 45° from each chair, a white cardboard display held 9 printed pictures of animals arranged in a 3 x 3 matrix. Two tilted cardboard stands, each facing one of the chairs, were put up on the table roughly at face height to hold participants’ notes for the conversation. Prior pilot runs had shown that, compared to notes posted at table height, the current setup resulted in overall more stable gaze tracking due to fewer head movements and fewer gazes below the range of the eye tracking glasses. Two ‘Tobii Glasses 2’ mobile eye trackers were used to simultaneously capture the gaze of both participants. These trackers have a sampling rate of 100 Hz and a spatial accuracy of ~ 0.89° when the observers are seated [41]. Additionally, a Logitech HD Pro C920 webcam mounted above the participants was used to capture the scene. Further information on the pictures is available on request from the corresponding author (LH).

### 2.4 Procedure

Participants were randomly assigned to one of two classrooms where they were informed that they would receive a 30-minute training on animal biology. Only one training contained information that was later relevant to the ability to visually estimate the age of depicted animals, while the other training focused on trivia related to the animals. All participants then first completed, on their own, the ranking task for no more than 5 minutes while noting down what they thought the correct rank order was. They then completed a 3-item questionnaire (see S1 Appendix for questionnaire items) rating their self-perceived performance in the task. Each trained participant was then paired with a non-trained participant. In a separate room, participants were fitted with a mobile eye tracker and seated at the table as described above. Their notes were posted on the cardboard stand facing the respective participant. The eye trackers were calibrated at the beginning of this dyadic session before the instructions were read, as past work indicates that in some instances it is possible that the knowledge of being recorded by eye trackers can influence gaze behavior in social situations, and that this effect vanishes after a few minutes when participants are not reminded (e.g., by doing a calibration) that they are wearing the glasses [42, 43]. To help encourage collaboration, participants were then informed that the best-performing dyad in the subsequent task would receive a small monetary reward in the form of a gift card. They were then instructed to discuss for 4 minutes the strategies that they each used on their own when completing the task. For this discussion they did not see the pictures as they were hidden behind a cover. Subsequently, participants were instructed to complete the ranking task collaboratively, again within 5 minutes, and the cover concealing the pictures was removed. Sitting on swivel chairs, participants were free to rotate their bodies and for most of the time, were turned towards the display rather than facing each other. The experimenter told participants to start each of their conversations as soon as they heard the door close, then started the eye trackers remotely, clapped their hands once in front of all cameras for later video synchronization and then left the room thereby audibly closing the door. They returned when participants’ time was over first to uncover the pictures and the second time to end the experiment. Participants were allowed to complete the last sentences of their conversation before the experimenter knocked on the door and entered the room. Participants then completed another 3-item questionnaire on who they felt had relatively more expertise on the topic during the conversation (oneself or the conversation partner; see S1 Appendix). Finally, the experimenter debriefed the participants. This report focuses on the 5 minutes of the ranking task, in which participants were collaboratively ranking the depicted items. Our focus for data analysis lies on the dynamics of joint gaze and gestures towards objects of interest with respect to interlocutors’ conversational turns and different levels of training.

### 2.5 Data analysis

#### Behavior coding

Eye tracking recordings were first processed using Tobii Pro Lab [44] to export videos with an overlaid gaze cursor. Following coding recommendations by [45], the two eye tracker videos and the scene camera video were then merged and synchronized using Mangold INTERACT [46] and the audiovisual signal from the recorded clap. With the same software synchronized videos were then used to manually code ‘looking’, ‘pointing’ and ‘turn-taking’ behavior. For a detailed description of the procedures regarding the recording and processing of mobile eye tracking data with Tobii Glasses 2 and synchronizing and manual coding using Mangold INTERACT see [47], as their procedures were followed in the present study.

Gaze towards a picture was defined as starting with the first frame in which the gaze cursor entered the picture until the last frame before it left the picture (‘looking at’ and ‘gaze at’ are used interchangeably throughout the paper). Pointing towards a picture was defined as starting with the first frame where a forward movement of a hand, which ended in a pointing gesture towards a picture, was visible up until the first frame of a retracting movement of that same gesture. As we synchronized two 60 fps videos with gaze overlay with the 30-fps video from the scene camera, our resulting frame rate in the merged videos from all three cameras was 30 fps. Consequently, this was the temporal accuracy with which we synchronized videos and coded behavior. All behaviors were coded in a mutually exclusive and exhaustive manner [45]. For turn-taking this meant that it was always one of the two participants’ turn, with no gaps where it was “no one’s turn”. A more detailed description of the coding procedure is provided in the supplementary material.

In the present study we were not interested in individual or mutual face gaze or their cuing effects initiating joint attention but rather the dynamics of ongoing joint attention towards an external object of interest, namely task relevant items [48]. As we were interested in joint object directed gaze rather than face directed gaze, only gaze directed onto task relevant items was included in the analysis.

Two coders, both blind to the hypotheses, were first trained on how to code behaviors with two example dyads which were not used for data analysis. Interrater reliabilities for gaze, pointing and conversational turns from another dyad coded by both coders were all above a Kappa of 0.70 and therefore found satisfactory [49]. Coded data were exported from Mangold INTERACT as event data and transformed into categorical time series with a sampling rate of 10 Hz.

#### Cross recurrence quantification analysis

To assess the dynamics of coupling across behaviors, Cross Recurrence Quantification Analysis was used [CRQA, for a comprehensive overview and formal discussion see 50]. CRQA allows one to visualize and quantify the dynamics of recurring states within behavioral systems [51–53]. Similar to cross-correlation, which provides a linear function of the co-variation of two time series, cross-recurrence is a form of generalized cross-correlation, which allows one to model nonlinear interrelations [54]. When employed with nominal data, CRQA can be understood as an extended version of lag sequential analysis, focusing on co-occurrences of behavioral states rather than their transitional probabilities [45, 52]. By extracting higher level abstracted measures from a system’s states over time, CRQA serves to describe underlying non-linear system dynamics. The method has already been applied in several studies examining the dynamics of interpersonal coordination [6, 11, 13, 15–19, 29, 30].

In the present study, we employed nominal CRQA on combinations of nominal time series data of gaze and pointing behavior as described below using the R package ‘crqa’ [55]. We first constructed recurrence plots, and from those average diagonal cross recurrence profiles (DCRPs) [56]. Using MATLAB [57] we calculated the center of recurrence mass (CORM) for each plot, to infer leading and following dynamics [20, 53]. The following paragraph explains recurrence plots, DCRPs and CORM and how we leveraged them for the purpose of this study (see also S3 Appendix for a detailed explanation).

#### Recurrence plots

**Fig 2:**
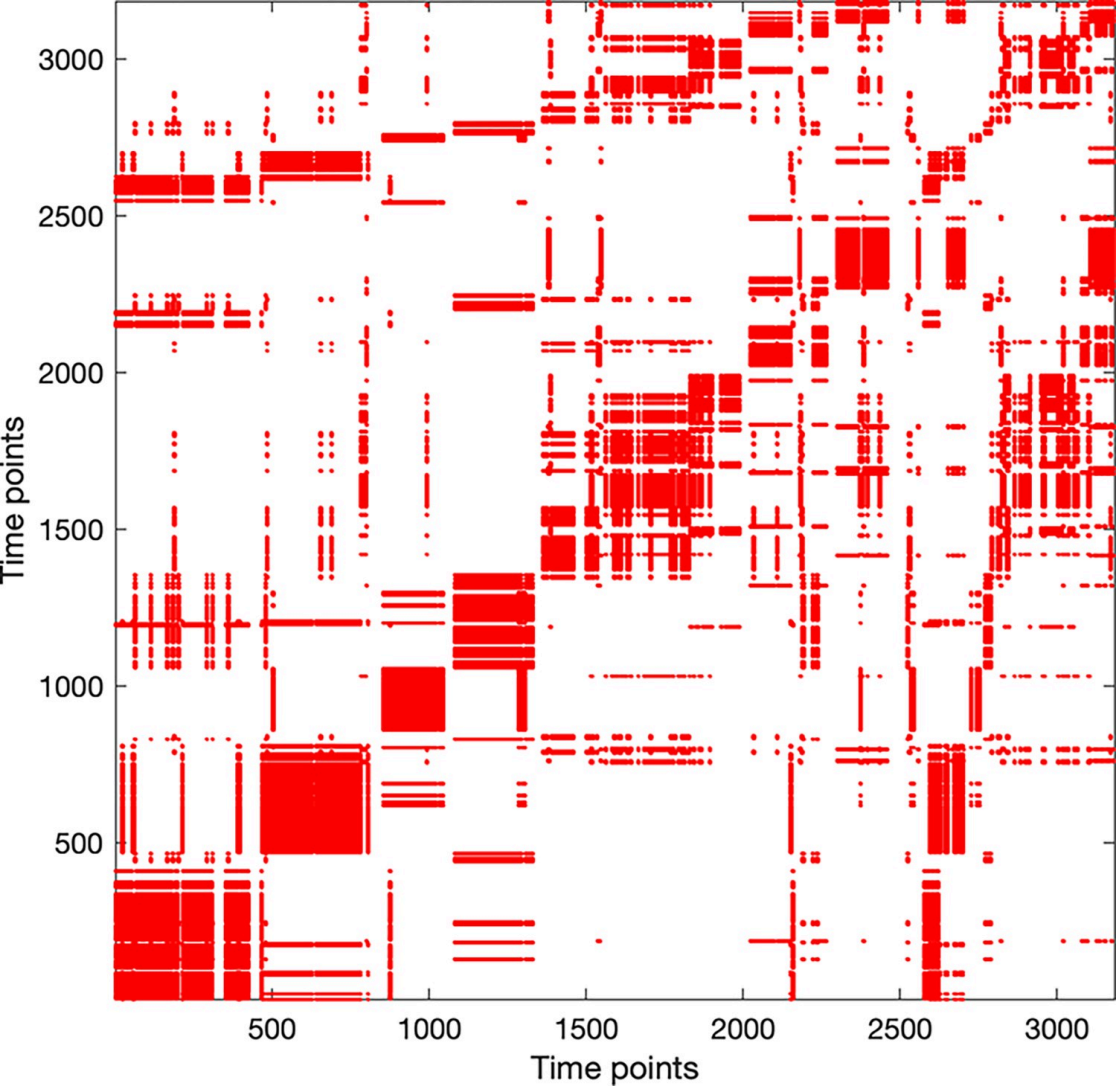
Example of a recurrence plot. Example of a recurrence plot constructed from nominal time series data of two interacting participants’ gaze towards the 9 task relevant items. The boxes along the diagonal line from the bottom left to the top right indicate moments of prolonged joint attention. Unique for this example dyad, symmetrical converging structures on the top left and bottom right, mirrored across line of coincidence indicate that both participants revisited all items during approximately the last minute of the conversation.

Recurrence plots allow one to capture coordinative structures in dyadic data over time (see Fig 2 for an example). They are a visualization of the pattern of co-occurrence of states that two streams of behaviors can take over time, and thus they represent coupling patterns of behaviors. In our case, recurrence plots show if both participants of a dyad were looking and / or pointing at the same item and, more importantly, “where in time” relative to one another they were doing that. Behaviors recurring closer together in time are plotted closer to the line of coincidence. This is the diagonal from bottom left to top right, corresponding to lag 0. If the same behavior is shown by one person, yet earlier or later in time relative to their counterpart’s behavior, this results in a recurrence point being plotted further away from the line of coincidence (i.e., at higher lags), towards the top left or bottom right corner, depending on which behavior leads the other. In our case, per dyad, we constructed recurrence plots using categorical time series data from combinations of looking, pointing and talking to address our hypotheses.

The example below (Fig 2) shows a recurrence plot from gaze data of one of the dyads of the present experiment. Blocks centered on the line of coincidence from bottom left to top right show that there are clustered incidences of closely coupled joint visual attention followed by brief moments of uncoupling. By qualitatively comparing recurrence plots with the recordings we find that as in the dyad whose data we see in Fig 2, it was a common strategy to simultaneously focus on the same item for some time before jointly switching to the next one. The more solid a block is, the fewer incidences of decoupling happened during that period. Short incidences decoupling often stemmed from only one of the two participants visually comparing the current item of interest with another one. While resulting patterns give a descriptive idea of a single system’s dynamics, more importantly, one can extract secondary plots, such as DCRPs and the CORM measure. These allow for inferential hypothesis testing across systems.

#### Diagonal cross recurrence profiles

**Fig 3:**
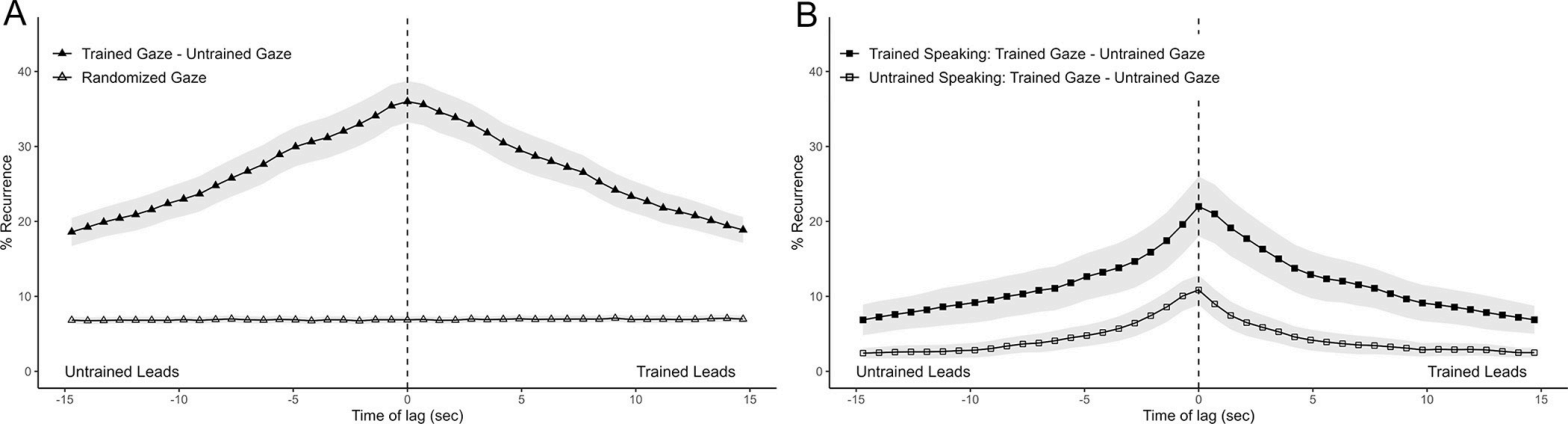
Average Diagonal Cross Recurrence Profiles (DCRPs) of gaze behavior between participants. (A) Average DCRP of trained participants looking and untrained participants looking. Ribbons represent standard error. Recurrence on positive time lags indicates that the trained participant’s behavior (looking) leads the untrained participant’s behavior (looking) in time. (B) Average DCRP from the same data, now split by participant’s conversational turn.

**Fig 4:**
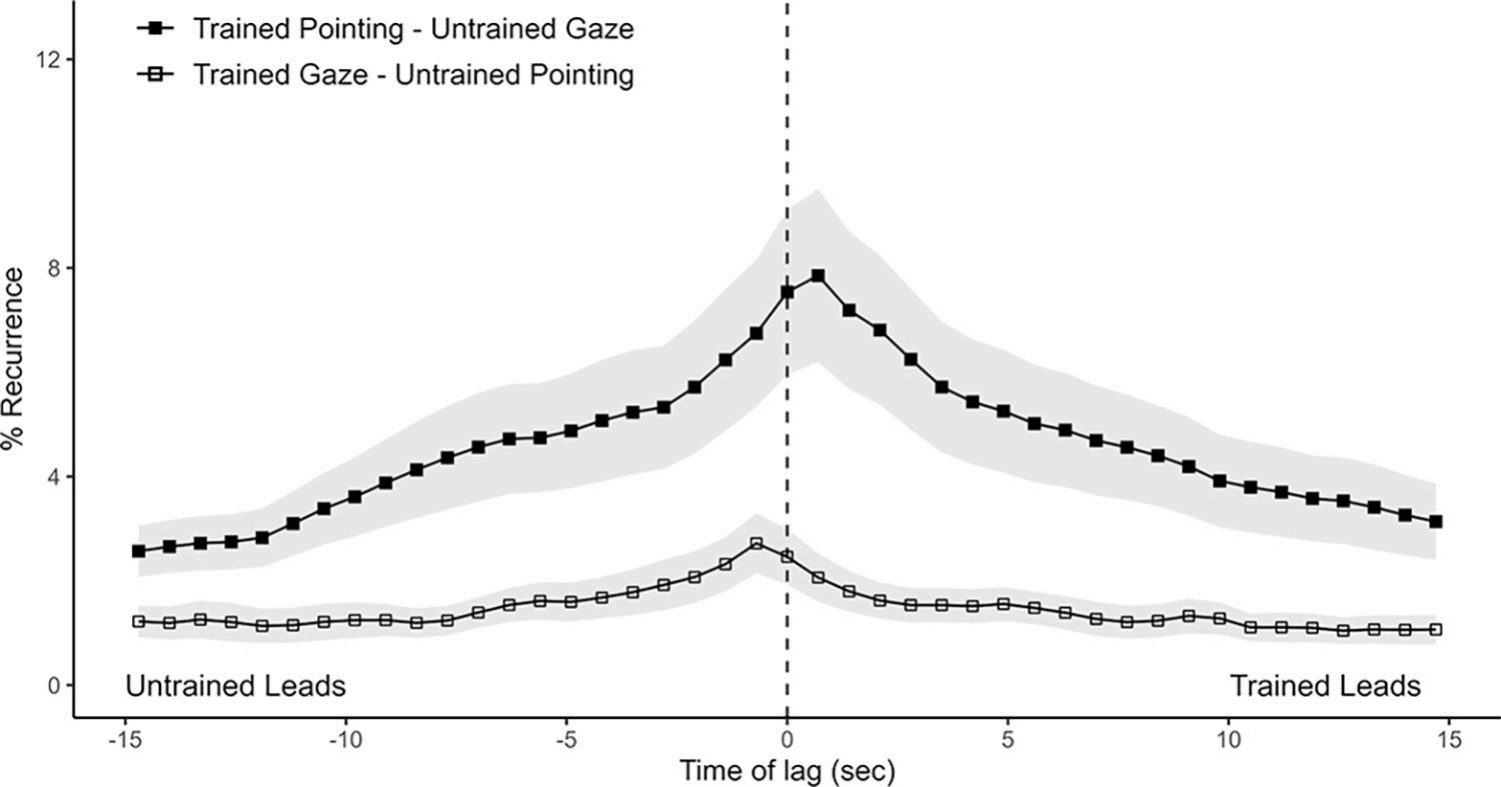
Average Diagonal Cross Recurrence Profiles (DCRPs) of pointing and gaze behavior between participants. DCRPs of participant’s pointing and their respective counterpart’s looking, split by training level. Ribbons represent standard error. Recurrence on positive time lags indicate that the trained participant’s behavior (looking or pointing) is leading in time.

**Fig 5:**
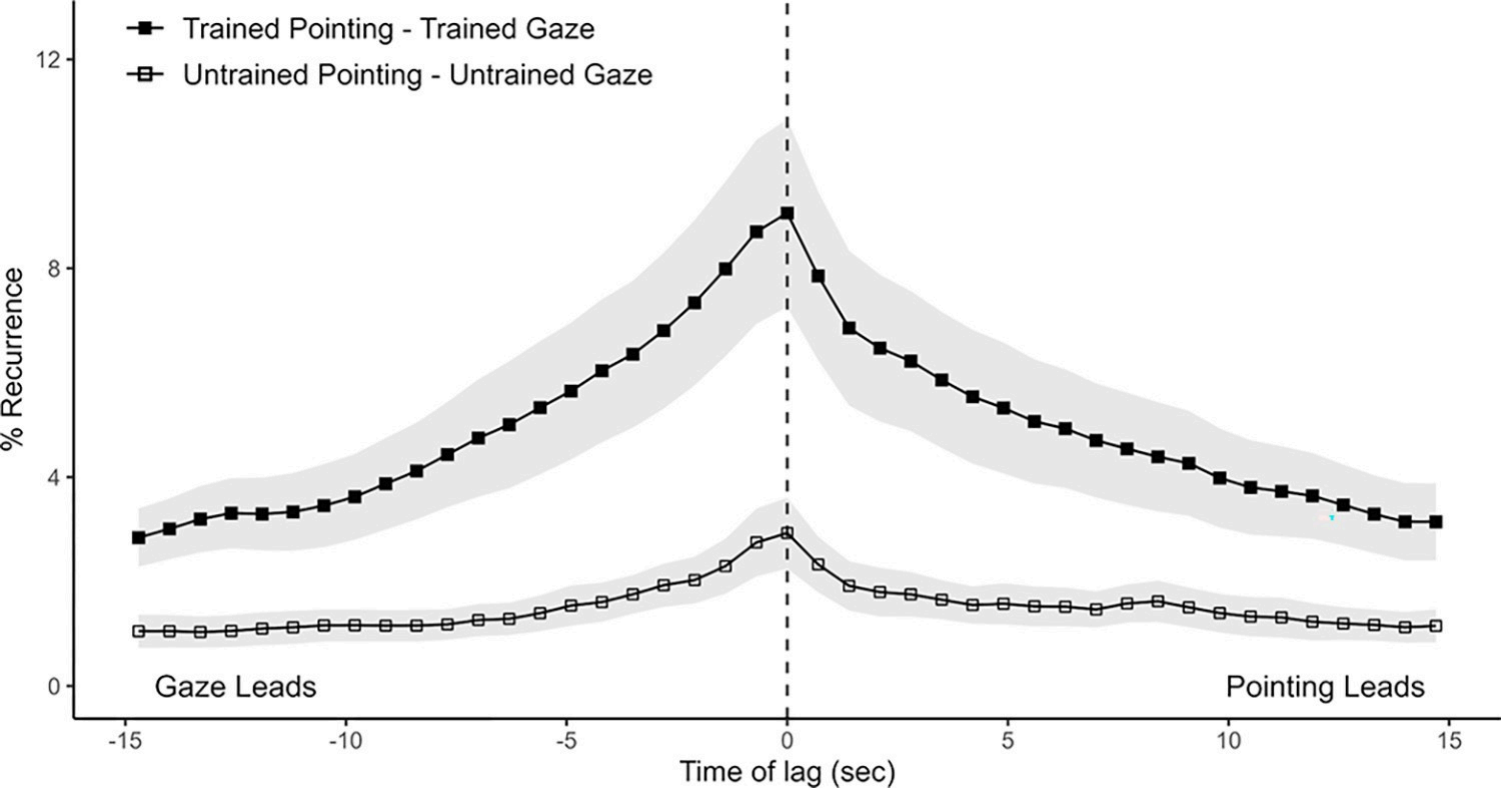
Average Diagonal Cross Recurrence Profiles (DCRPs) of pointing and gaze behavior within each participant. DCRPs represent participants’ pointing and their own looking, split by training level. Ribbons represent standard error. Note that this plot is constructed differently to the earlier ones as the DCRPs depict coupling of behavior within the same participants rather than across a dyad. Recurrence on positive time lags here indicates that pointing leads looking, whereas those on negative time lags indicate that looking leads pointing.

From each recurrence plot we constructed a DCRP. These plots capture the proportion of recurrence points (recurrence rate) for a range of lags, centered at time lag 0. The recurrence rate (see S3 Appendix) is one of the simplest measures to operationalize an amount or strength of coupling as it describes the probability of co-occurrence of two behaviors within some specified time window. Average DCRPs are thus each depicting behavioral dynamics of a combination of gaze and/or pointing and are shown in Figs 3–5. Each figure thus shows the average coupling dynamics for one pair of behaviors across all conversations. This representation of average recurrence rates as a function of lags around the line of coincidence allows one to see which of two behaviors leads in time and at what lag the two behavioral streams are coupled the most. If the plot is mirrored symmetrically along the central-y axis (lag 0), there is on average no leading or following of behaviors. An asymmetric plot though, with a peak offset from lag 0, indicates one behavior lagging behind the other one, mostly so at that lag in time [see 56 for a visual illustration and explanation and S3 Appendix on how DCRPs are constructed from recurrence plots]. In our case, the range of delays (positive and negative) taken into account for constructing DCRPs was set to 150 time points, corresponding to 15 seconds of lag in either direction. Evenly aggregating the overall 301 time points of lag resulted in 43 bins to be considered for analysis. To be informative, this distribution must be set in relation to a randomized baseline, that shows which base level of recurrence rate one would expect for this system at each lag if the underlying time series were not coordinated. This can be achieved by either running the analysis with a randomly paired dyad or by shuffling timeseries data within dyads, which removes the temporal structure of the data while maintaining the base chance of recurring data points. We had to use the latter approach of shuffling data, as conversations across dyads were not all of the exact same length, therefore we could not create mismatched pairs. A lag range of +/- 15 seconds for the DCRPs was chosen as this was the approximate range in which all behaviors showed to be coupled above chance. Also, during cooperative interaction, dyadic gaze coordination [19] and dyadic eye-hand coordination [30] have shown to be meaningfully coupled within similar time windows.

#### Center of recurrence mass

As we established, a DCRP peaking above or below lag 0 indicates one behavior lagging behind the other one on average. Yet even when two behaviors mostly coincide in time, and the DCRP thus peaks at lag 0, the plot can be asymmetric with regard to the central y-axis (lag 0). This then also indicates leading / following between behaviors and can be quantified by the center of recurrence mass (CORM) of the coupled behaviors. This measure has been brought forth by Anderson and colleagues [53] and is defined as the normalized distance of the center of gravity of recurrent points from the line of coincidence. CORM thus approximates on which side of the line of coincidence the majority of recurrence points on a recurrence plot are situated. It therefore serves as a quantified value that indicates which time series leads the other, regardless of the characteristics of the DCRP, such as the peak. The sign of the value indicates the direction of the effect and the absolute value its size (see S3 Appendix for a detailed explanation of CORM). As we were interested in the temporal dynamics of leading and following from moment to moment (i.e., close to the line of coincidence) rather than globally for the whole recurrence plot, we calculated CORM including recurrence points within a band of +/- 4 seconds temporal lag. We chose this time window as judged by the shape of constructed DCRPs, subtle turn dependent differences in gaze dynamics seemed to take place within those small lags in time. So this banded CORM measure is a way of “zooming into” temporally more nuanced effects compared to the more broadly captured effects of the DCRPs. For each system, average CORM values across dyads were calculated and used for analysis [53].

For further details on DCRPs, CORM, and CRQA in general, see [20, 55, 56, 58]. For all analyses, prerequisite assumptions were tested prior to conducting parametric tests. In instances where these were not met, we employed the non-parametric equivalent. The data underlying the results presented in the study are available from https://osf.io/7bs6y/.

## 3. Results

Before conducting the main analyses, we were interested whether participants were aware of their own (relative) expertise. Specifically, if they self-rated in accordance with their training with regard to (A) their own performance on the task they completed alone, and (B) their relative expertise during the conversation (see S1 Appendix for questionnaire items). A two-sample t-test on participants’ self-perceived performance ratings on the task they first each completed on their own indicated a marginally significant effect of trained participants self-rating their own performance higher (M = 3.26, SD = 0.77) than untrained participants rated their own performance (M = 2.74, SD = 0.82) on a 6-point Likert scale, t(34) = 1.94, p = 0.060, 95% CI [-0.024, 1.061]. Further, a paired t-test on participants’ ratings on who had relatively more expertise during the conversation indicated trained participants’ ratings to be significantly higher (i.e. closer to “I had more expertise.”, M = 5.16, SD = 0.68) compared to untrained participants’ ratings (closer to “My partner had more expertise.”, M = 2.00, SD = 0.78) with a mean difference of 3.16 on a 7-point Likert scale, t(17) = 11.51, p < 0.001, 95% CI [2.59, 3.75]. These results indicate that while there was only a tendency of participants to be aware of their own expertise before the conversation, talking to their partner made them aware of their differential expertise. This is supported by anecdotal evidence from the audio recordings, where some participants indicated that there might have been a difference in the training they received or their ability to remember relevant information from the training. Thus, we suggest that participants were aware of their own expertise to some extent before the conversation, and definitely afterwards, which supports the effectiveness of the training manipulation.

### 3.1 Looking and speaking

#### H1: Time spent speaking

We then first tested whether the introduction of differential knowledge had the expected effect on the amount of talking participants showed, as according to H1, people with more objective and perceived competence should talk more [36]. Results show that the average conversation took approximately 5 minutes and 14 seconds (SD = 22.2 sec). Trained participants spoke longer (median = 3 min, 0 sec, median absolute deviation (MAD) = 35.5 sec; 59% of the conversation time) than their counterparts (median = 2 min, 16 sec, MAD = 42.4 sec; 41% of the conversation time), Wilcoxon W = 275, *p* < .001. This confirms our first hypothesis.

#### H2: Trained gaze and untrained gaze

We hypothesized asymmetric gaze coupling between dyads, specifically that the trained participants lead with their gaze relative to the gaze of the untrained participant (H2). To test this, we first generated cross-recurrence plots for each of the 18 dyads. Fig 2 above shows the recurrence plot of one dyad’s gaze data. To address the question of whose gaze leads during the conversation, we generated DCRPs for each recurrence plot, which capture the percentage of recurrence in each dyad for each time lag, centered on time lag zero. Fig 3(A) shows the average DCRP for all dyads within a +/-15 seconds lag window. As mentioned in the Methods section, this distribution must be set in relation to a randomized baseline, which shows which base level of recurrence rate one would expect for this system at each lag. Fig 3(B) shows this baseline, namely the randomized average DCRP for trained and untrained participant’s gaze.

A 2 (profiles; gaze vs. randomized baseline) × 43 (lags) mixed-design analysis of variance (ANOVA; with lag as the repeated measures factor) revealed a main effect of gaze compared to randomized baseline *F*(1, 731) = 3608.31, *p* < .001, indicating that interlocutor’s gaze was coupled well above chance level within the +/- 15 sec window. Even though coupling seemed substantially high above chance level beyond these marks, we did not take further lags into account as we were interested in the moment-to-moment properties of the conversation rather than the overall coupling within a dyad. There was also a main effect of lag *F*(42, 714) = 94.23, *p* < .001 and an interaction effect between profiles and lags, *F*(42, 731) = 6.302, *p* < .001), suggesting that the level of lag influenced cross-recurrence overall and that the difference between the profile versus the randomized profile varied across different levels of lag. As visible by the peak of the DCRP in Fig 3A, a maximum recurrence rate of 36% occurred at lag 0. This indicates that dyads mostly looked at the same item at the same time and did so for 36% of the conversation. Contrary to our first hypothesis, this central peak and the symmetry of the DCRP indicates that on average (over the course of the 5-minute conversation), neither side led nor followed more with their gaze than the other. This is supported by the average CORM value across dyads, which is not different from zero (M = 0.01, SD = 0.03, CI [-0.007,0.026]), as indicated by a one-sample t-test, t(17) = 1.23, p = 0.237. We thus fail to support the second hypothesis, that trained participants lead with their gaze relative to the gaze of the untrained participant.

Before continuing with further results, for the sake of expediency, we note here that all of the following reported DCRPs were found to be coupled at above chance levels using mixed-design ANOVAs. They also all show the same interaction pattern as reported above when analyzed with their randomized baseline. This means that all DCRPs we investigated were coupled at above chance levels and to varying degrees across lags. This is reported in more detail in the supporting information.

#### H3: Trained gaze and untrained gaze. Split by conversational turn

We hypothesized that asymmetries in gaze coupling might be dependent on conversational turn-taking, specifically that whoever speaks also leads the dyad’s joint focus of attention at those moments (H3). To test this, we repeated the above analysis, except on this occasion we created two sets of time series from the initial one depending on who was speaking at any given moment. We thus constructed two sets of 18 recurrence plots and consequently two average DCRPs, as seen in Fig 3B (see S3 Appendix for a detailed explanation).

We compared the two profiles from split data across time lags with a 2 (turns) x 43 (lags) repeated-measures ANOVA. This revealed a main effect for turn, (*F*(1, 17) = 11.99, *p* = .003), indicating higher recurrence values when trained participants were speaking as well as a main effect for lag (*F*(42, 714) = 110.48, *p* < .001), suggesting that overall recurrence rate varied across different time lags. The interaction effect for lag and turn (*F*(42, 714) = 13.05, *p* < .001) further suggests that the difference in recurrence between turns did vary across the +/- 15 seconds of time lag. As we established earlier, trained participants spoke quite a lot more than untrained participants. The main effect of higher gaze recurrence for when trained participants are speaking is a product of this and thus expected. Interestingly, and in line with the interaction effect, there seems to be a difference in shape between the two DCRPs, notably mirrored along the central y-axis. Specifically, the upper plot’s center of mass seems to be shifted to the right, where recurrence points indicate trained (currently speaking) participants’ gaze leads in time. In contrast, the lower plot’s center of mass seems shifted to the left, where recurrence points indicate untrained (currently speaking) participants’ gaze leads. A differential center of mass between these plots would indicate an asymmetry in gaze coupling dependent on conversational turn. Thus, we tested for a difference in CORM between the two DCRPs.

A paired t-test indicates higher CORM values for gaze coupling while trained participants were speaking (M = 0.03, SD = 0.03), compared to when untrained participants were speaking (M = -0.03, SD = 0.05), (*t*(17) = 3.682, *p* = .002). Further, one sample t-tests indicate CORM values differing from zero while trained participants were speaking (*t*(17) = 4.410, *p* < .001) as well as while untrained participants were speaking (*t*(17) = -2.1854, *p* =. 043). This means that the momentary speakers’ gaze leads momentary listeners’ gaze, despite the DCRP peaking at lag 0.

Taken together, within +/- 15 seconds of lag, the eye movements of the two conversation partners were linked above chance level, mostly so at lag 0, indicating that participants looked at the same item at the same time for most of the time. Contrary to our hypothesis (H2), we did not find an indicator of asymmetric gaze coupling—that is, trained participants did not lead with their gaze overall. Only when we analyzed gaze coupling with respect to conversational turn taking, were asymmetries revealed. Based on ANOVA results and the CORM, whoever’s turn it is to talk, leads the dyad’s joint focus of attention. Further, we saw that beyond the back and forth of conversational turns, attentional coupling still steadily peaked at lag 0. This means that while participants’ gaze was mostly coupled as they concurrently look at the same item at the same time, currently speaking participants’ gaze transitioned items earlier with respect to their listening counterparts’ gaze.

It is worth noting that the recurrence rates in the two resulting DCRPs (Fig 3B) do not add up to the sum of recurrence rates from the earlier DCRP comprising the whole dialogue, indiscriminate of conversational turn taking (Fig 3A). This is expected, as in a recurrence plot with gaze data from the whole dialogue, gaze can recur beyond the back and forth of conversational turns, hence leading to more recurrence than the sum of recurrences from two recurrence plots from gaze data split by conversational turn does.

In the next section, we present findings regarding the dynamics of looking and pointing across participants (i.e., how one person points and the other one looks) and within participants (i.e., how one participant points and the same participant looks). Note that participants almost only pointed while speaking, making an analysis regarding pointing and speaking unnecessary.

### 3.2 Looking and pointing

#### H4: Time spent pointing

In keeping with H1 (people with more objective and perceived competence talk more), we tested if people with more objective and perceived competence pointed more [e.g., 36]. Results show that trained participants on average pointed more (12% of the conversation time) than untrained participants (4% of the conversation time), Wilcoxon W = 234, *p* = .024, confirming H4.

As noted previously, there are several outstanding unknowns regarding the fine-grained dynamics between gaze and pointing during an ongoing interaction. For example, where in time between one’s own focus of attention and an interlocutor’s focus of attention is pointing situated? Below we present and test several explorative questions.

#### Is pointing and gaze between participants coupled symmetrically or asymmetrically?

To address this question we conducted an analysis that was conceptually identical to one that we applied previously to the participants’ gaze data, only that this time, we conducted the analysis twice, once with one person’s gaze data and their interlocutor’s pointing data, the other time with the relationship reversed: (a) trained pointing and untrained looking; (b) trained looking and untrained pointing (see Fig 4 for an illustration of the data).

Two of the untrained participants did not point at all, so their dyads were excluded from analysis. A 2 (profiles; trained pointing & untrained looking vs. trained looking & untrained pointing) x 43 (lags) repeated-measures ANOVA revealed a main effect for profiles (*F*(1, 15) = 5.55, *p* = .032), indicating higher recurrence rates when trained participants pointed and untrained participants looked than vice versa. This is in line with what we would expect if, overall, more time is spent pointing by trained participants than untrained participants. Further, a main effect of lag (*F*(42, 630) = 15.13, *p* < .001) and an interaction between lags and profiles (*F*(42, 630) = 4.85, *p* < .001) indicates that overall recurrence rates varied across different time lags though not between profiles. This is in line with what we see on the plotted DCRPs in Fig 4. They show peaks on different sides from lag zero in a manner that suggests that one participant’s pointing leads the other’s gaze by roughly 500 ms.

The analysis of the CORM profiles returns significantly different CORMs between profiles (trained pointing–untrained gaze: median = 0.04, MAD = 0.05; untrained pointing–trained gaze: median = -0.03, MAD = 0.08; *V* = 117, *p* = .009), indicating that one person’s pointing leads the other person’s looking significantly. This result also underlines that whoever is pointing at the time is leading the other person’s gaze. Does the pointing—gaze asymmetry extend to one’s own gaze and pointing?

#### How are one’s own gaze and pointing coordinated?

To answer this question, the same analysis regarding pointing and gaze was conducted as above, with the difference being that this time, it was conducted with gaze- and pointing data from the same person (Fig 5). Note that as the two behavioral time series refer to the same person, the recurrence plots, and therefore the DCRPs, need to be constructed in a manner where positive or negative lags reflect the relationship between different behaviors rather than individuals. Accordingly, in Fig 5 recurrence at negative lags (left side) indicates that looking leads pointing, while those at positive lags (right side) indicate that pointing leads looking.

The two resulting DCRPs, together with a 2 (profiles; trained pointing & trained looking vs. untrained pointing & untrained looking) x 43 (lags) repeated-measures ANOVA returned significant main effects for profile (*F*(1, 15) = 5.71, *p* = .030) and lag (*F*(42, 630) = 20.85, *p* < .001), and a significant interaction (*F*(42, 630) = 5.97, *p* < .001). This indicates that there are higher recurrence rates for trained participants looking at their own pointing, and that recurrence rates varied across different time lags and between profiles. Regardless of training the recurrence rates peak at lag 0.

Note that both trained and untrained DCRPs seem to be asymmetric with respect to lag 0. We found that their CORM is also shifted to the left, which indicates that the probability of participants looking at something before they point at it is higher than the reverse. CORM analysis reveals that while mean CORM is indeed negative for both profiles, statistical tests against zero reveals this effect to be significant only for the untrained participants (V = 20, p = .011, median = -0.04, MAD = 0.07, 95% CI [-0.157, -0.007]); trained participants (V = 34, p = .083, median = -0.01, MAD = 0.06, 95% CI [-0.086, 0.006]). Point estimates and confidence intervals of untrained and trained CORM though suggests compatible effects [59] with one’s own gaze preceding one’s own pointing in time more often than not. We thus cautiously interpret these analyses as suggesting that one’s own gaze precedes one’s pointing regardless of training, though this may be more pronounced for the untrained participants. However, we hasten to note as well that in general, as there are far more and longer incidences of participants looking at pictures than them pointing at pictures, the results regarding pointing lack the robustness of those regarding gaze.

## 4. General discussion

The aim of the present study was to examine how interpersonal coordination of attention is achieved through multimodal coupling of gaze and deictic gestures during non-scripted dialogue. We manipulated task-relevant background knowledge of participants who then jointly solved a visual sorting-task while being co-located, wearing mobile eye trackers, and being able to freely gesture and converse with one another. Nominal cross recurrence quantification analysis (CRQA) was used to assess behavioral coupling between participants, and leader-follower roles, overall and during conversational turn-taking.

There were four main findings, two regarding the symmetry of gaze coupling and two regarding the symmetry in the coupling of deictic gestures and gaze. Below we review these results and elaborate on their implications for the study of attentional coordination.

### 4.1 Symmetry of gaze coupling

There were two main findings regarding the symmetry of gaze coupling. First, contrary to our hypothesis that trained individuals lead with their gaze in relation to their untrained counterpart’s gaze (H2), we did not find such asymmetry in gaze coupling. So despite the fact that participants were likely aware of a difference in expertise, and in line with our hypotheses that trained participants talked (H1) and pointed (H4) more, neither trained nor untrained participants consistently led the conversation with their gaze. Second, regardless of training, gaze data split by conversational turns revealed that in line with our hypothesis H3 (speakers lead the listeners’ gaze) gaze was coupled asymmetrically between participants insofar that whichever participant was momentarily speaking, this individual led the dyads joint focus of attention at those moments. Together, the data indicate that both participants lead with their gaze when it was their turn to talk, regardless of their training.

#### Gaze coupling and differential knowledge

While other studies have found asymmetries in attentional coupling between interlocutors with predefined differential conversational roles [15, 17, 18], we could not replicate this effect between interlocutors with differential knowledge. Contrary to our expectation of trained participants leading with their gaze (H2), both participants typically looked at the same item simultaneously without any detectable coupling asymmetries (e.g., the person with more knowledge did not tend to lead the person with less knowledge). So even though differential knowledge led to trained participants dominating the conversation by speaking and pointing more, this domination was not reflected in asymmetric gaze coupling.

In fact, the symmetry of gaze coupling we found is comparable to the one reported for gaze coupling in natural dialogue without any assigned roles [19]. Further, while we know that the amount of knowledge interlocutors share (matched vs. mismatched) affects how highly (symmetrically) coupled their gaze becomes [19], our results show that differential relevance of knowledge between interlocutors does not necessarily affect the symmetry of gaze coupling between them.

So how is it that one interlocutor dominates the conversation by talking and pointing more, yet both interlocutors are still mostly looking at the same item at the same time? One reason for this could be that, unlike the studies that found asymmetric attentional coupling using set conversational roles such as “speaker” and “listener” [18] or “Director” and “Matcher” [17], in our study participants had relatively more freedom in how to approach the conversation. They could freely take turns talking about, and pointing at, items in ways that benefited them to become and stay coupled with their attention. Given the idea that higher gaze coupling can be beneficial for communicative success [13], this behavioral freedom together with a shared motivation to optimize their performance could have enabled dyads to coincide their gaze performance so strongly that the differential amount of speaking and pointing did not carry enough weight to break this symmetry.

#### Gaze coupling and conversational turns

In line with our hypothesis that the momentary speaker leads the focus of attention (H3), when we investigated gaze data split by conversational turns, gaze was coupled asymmetrically. As indicated by the CORM, whichever participant was momentarily speaking tended to lead the dyads’ joint focus of attention. Notably, this was true despite gaze always being coupled most strongly at lag 0, hence individuals still mostly gazed at the same item at the same time regardless of conversational turns. Only the probability of who transitions to the next item earlier was affected by conversational turns. So participants’ gaze was continuously coupled, yet in tune with the conversational back and forth of turn taking, asymmetrically. This effect prevailed regardless of training. Two conclusions can be drawn from this.

First, earlier studies show symmetric coupling in dialogue [19], yet asymmetric coupling during monologue, where listeners’ gaze lagged behind speakers’ gaze by approximately 2 seconds [18]. Our finding extends those earlier findings by showing that there is indeed asymmetric gaze coupling during dialogue, yet it is only found when discriminating between conversational turns. This shows that for the study of attentional coordination, it can be a fruitful approach to discriminate in which conversational state each actor is at each given moment—that is, that of the information sender (i.e., speaker) or the information receiver (i.e., listener). Aggregating data across such moments can mask the momentary asymmetries in dynamics between them. We also show that, next to the analysis of features of the DCRP, the use of the CORM can elucidate such subtle asymmetries.

Second, because trained participants spent more time in the conversational role of the speaker, they led with their gaze for longer than untrained partners. This ties in nicely with earlier findings that underline the importance of speaking time in social interaction: More competent individuals talk more and are rated as more competent partly due to the fact that they are talking more [36]. We show that whoever talks more also determines more where a dyad’s coupled focus of attention is directed. In a broader sense this asymmetric attentional coupling can be seen as a behavioral facet of “infocopying”, a form of social learning, from a more skilled individual to a less skilled one [33]. From the recordings we know that trained participants often used their speaking time to state key information that (deliberately) was missing from their counterparts’ training. In a task where both individuals are interested in performing well together and know who has superior expertise, it makes sense to freely confer more speaking time to the more knowledgeable individual. Our study shows that this tendency is also reflected in the coordination of attentional coupling that allows both interlocutors to profit from one person’s superior knowledge.

We conclude that differences between co-actors’ task relevant knowledge during dialogue does not lead to asymmetric gaze coupling such that the more knowledgeable person overall leads with their gaze, at least when investigating the average gaze dynamics for the whole conversation. Only when taking turn dependent asymmetries into account does it become apparent that whoever momentarily speaks also leads dyadic attentional coupling through earlier gaze transitions between items. In that sense, in the present study, more knowledgeable individuals did lead with their gaze for longer stretches of time as they were speaking more.

### 4.2 Coupling of deictic gestures and gaze

There were two main findings regarding the symmetry of the coupling of deictic gestures and gaze. First, we found that pointing and gaze were coupled asymmetrically across participants insofar that gaze lagged behind others’ pointing by approximately 500 ms. Second, pointing and gaze within participants tended to be asymmetrically coupled as one’s own gaze tended to precede one’s own pointing, even though they were mostly recurring simultaneously. Together, this implies that pointing serves as a mediator of attention from the pointing person to their interlocutor. Below we discuss implications of these findings.

#### Coupling of gaze with others’ pointing

Pointing and looking across participants was asymmetrically coupled above chance and in a manner that indicated that one participant’s gaze lagged behind the other participant’s pointing by approximately 500 ms. These coupling dynamics prevailed for both participants regardless of training, even though trained participants pointed more than untrained participants. Interestingly, the same pattern emerges as with gaze coupling and turn taking. While the amount of coordinative behavior (in this case pointing) shown per participant is affected by their training, the attentional dynamics between participants remain unaffected. On average, an untrained participant’s pointing gesture is followed by the trained participant’s gaze just as quickly as in the trained pointer—untrained gaze relationship. Together this suggests stable attentional dynamics that do not depend on differences in participants’ minds. Thus, trained participants did not dominate the conversation because their pointing gestures elicited different coupling dynamics with their counterpart’s gaze, but simply because they pointed roughly three times as much as the untrained participants. Importantly, participants almost solely pointed while speaking. Hence, asymmetries for gaze-gaze coupling and hand-gaze coupling are observed as both are in tune with the conversational back and forth of turn-taking. This underlines the notion that asymmetries in non-verbal behavioral dynamics between conversing interlocutors can be masked by aggregating data across relevant moments, such as a switch in conversational turns.

As noted in the introduction, earlier studies have highlighted the nonverbal role of hands in attentional coordination [29–31]. For instance, the coordination between an infant’s gaze at their caretaker’s hands and vice versa has been shown to be a viable avenue to achieve joint attention between them, rather than the classically investigated route of ‘gaze-gaze’ coordination [30, 31]. These studies demonstrate that hand movements convey attentional meaning, and that the coupling of gaze with those movements can serve as a measure of joint attention. Our study extends these findings. We show how eye-hand coordination across adult interlocutors who exchange information from one conversational turn to the other can also serve as a valuable tool to become and stay coupled with each other’s attention. Crucially, we further show that the dynamics of this coupling are dependent on conversational turn-taking.

#### Coupling of gaze with one’s own pointing

One’s own pointing and gaze was asymmetrically coupled in such a manner that the probability of participants looking at an item before also pointing at it was higher than the probability of participants pointing at an item before also looking at it. Notably, gaze and pointing gestures towards the same item mostly co-occurred in time. Only the probabilities differed as to which behavior came first, gaze or gesture, before the respective other joined in. Overall, this effect proved to be less robust than those reported for gaze and thus it should be interpreted with a degree of caution.

While we already discussed statistical reasons for why the effect of one’s own looking leading one’s own pointing was significant solely for the untrained individuals, there are other possibilities. For example it is possible that trained participants know better what features they need to look for in the items in the environment and what those features mean. This could make the whole process of finding and conveying information less demanding, leading to their eyes to become more aligned in time with their pointing gestures. In any case, speculations like these provide exciting hypotheses for follow-up studies.

In sum, it appears that pointing serves as a mediator between ones’ own and others’ visual attention. In our study interlocutors point at an item while mostly simultaneously looking at it (with a tendency to already having looked at it earlier) and their counterpart on average looks at that item lagging behind in time by roughly 500 ms. To provide a reference, in Richardson and Dale’s study [18] with set speaker and listener roles, speakers on average fixate an item (in their case character portraits from a television series) 800 to 1,000 ms before naming it and listeners fixate an item 500 to 1,000 ms after the onset of the speaker naming it. So pointing during conversation in our task seems to lead to a slightly tighter mediation of attention than spoken words alone do in Richardson and Dale’s study [18]. Naturally, there is more to the coupling dynamics of attention than where in time coupling peaks and the effects from those two different tasks cannot be compared one to one. However, this way of comparing attentional coupling within and across modalities can provide rough estimations about the time spans in which people coordinate each other’s attention in space.

### 4.3 Limitations and future directions

A notable consideration is that task-specific affordances constrain how the interpersonal coordination of people under those constraints can unfold [12]. As task constraints get more difficult, interpersonal coordination increases and becomes more stable [11, 15]. In our case there were two notable constraints, adding to task difficulty. One, participants had to discuss quite complex visual details not only between items, but also elaborate them within items, meaning they had to be focused on the same item for stretches of time. This alone leads to increased recurrence rates compared to tasks where a more global visual approach (e.g., visual search) is beneficial. Two, the 5-minute time limit in which participants had to complete their task together made them very focused on completing the task. Participants spent their time highly engaged with the items (e.g., there was little irrelevant discussion), leading to relatively high amounts of time spent looking and pointing. Both of these factors likely contributed to relatively high attentional coupling. A future approach to investigate interpersonal coordination of attention could be to vary task constraints, like item complexity or time to complete the task, similar to Miles and colleagues [11], who varied the level of background noise during dyadic conversations to investigate the adaptive coordination of communicative behaviors across conversants. Moreover, in our study participants readily used pointing gestures together with deictic verbal expressions to identify the location of target items (e.g. “*This upper one*.”) but also for feature descriptions within items (“*The color here is quite dark*.”). Further, pointing gestures were used for different epistemic reasons, such as asking for clarification or providing clarification. In all these use cases, the pointing hand helps to disambiguate the current focus of attention and thus reduces overall verbal effort [4]. Future studies could manipulate how much participants are able to use their hands to elucidate and disambiguate information.

While the present work has addressed the role of conversational dynamics on attentional coupling between items, whether the same principles apply within items is unknown. In recurrence plots like the one in Fig 2, the prevalence of solid blocks around the line of coincidence shows that dyads spent substantial amounts of time looking at the same item together while transitioning to other items from time to time. From the recordings we know that participants showed this behavior to discuss features within the same picture while occasionally comparing them to features from other pictures. As attention is often object-based [60], preferring to move within rather than between objects [61], this promises to be a fruitful area of research for future investigation.

Another future direction would be to take others face and gaze direction into consideration. Pointing seems to be the “more dominant” deictic cue compared to face and gaze direction when investigated in an experimentally controlled design using a cartoon figure to cue attention [62]. However, the face of a pointing person is still often looked at, even in situations where the pointing gesture alone provides more reliable deictic information [25]. In our experimental setup, participants did not face each other, but sat next to each other, facing the items. A design where participants can see each other’s faces could enable the experimental examination of the relative contributions of face, gaze, and deictic gestures in interpersonal attentional coordination.

## 5. Conclusion

This study is one of the first to incorporate measures of both gaze and deictic gestures to investigate multimodal coupling of attention between adult conversants. It shows how interlocutors use their eyes and hands to dynamically coordinate attention in a relatively unconstrained, dyadic interaction. Previous studies have found that attentional coupling is temporally asymmetric when dyads are assigned conversational roles. In contrast, the present study investigates if this effect replicates in natural conversation where asymmetry is not an inherent part of the task itself but tied to differences that each individual brings to the task, specifically, their background knowledge. Using CRQA, we find that the coupling dynamics, both for participants’ gaze and gestures, are asymmetrically in tune with the alternation of conversational turns, yet independent of the higher amount of talking and pointing shown by more knowledgeable individuals. Together, these findings suggest stable, turn-dependent interpersonal coupling dynamics regardless of background knowledge. By highlighting this role of deictic gestures and conversational turn-taking, the current work broadens the field’s understanding of how people coordinate their attention.

## Supporting information

S1 Appendix Training & self-rated expertise questionnaire items. (PDF)

S2 Appendix Behavioral coding rules. (PDF)

S3 Appendix Cross recurrence. (PDF)

S4 Appendix All DCRPs and ANOVA results. (PDF)

## References

[1] Sebanz N, Bekkering H, Knoblich G. Joint action: Bodies and minds moving together. Trends Cogn Sci. 2006;10: 70–76. doi: 10.1016/j.tics.2005.12.009 16406326

[2] Stukenbrock A., Deixis Meta-Perceptive Gaze Practices, and the Interactional Achievement of Joint Attention. Front Psychol. 2020;11: 1779. doi: 10.3389/fpsyg.2020.01779 33041877 PMC7518716

[3] Todisco E, Guijarro-Fuentes P, Collier J, Coventry KR. The temporal dynamics of deictic communication. First Lang. 2021;41: 154–178. doi: 10.1177/0142723720936789

[4] Bangerter A. Using pointing and describing to achieve joint focus of attention in dialogue. Psychol Sci. 2004;15: 415–419. doi: 10.1111/j.0956-7976.2004.00694.x 15147496

[5] Ho S, Foulsham T, Kingstone A. Speaking and Listening with the Eyes: Gaze Signaling during Dyadic Interactions. Guo K, editor. PLOS ONE. 2015;10: e0136905. doi: 10.1371/journal.pone.0136905 26309216 PMC4550266

[6] Rohlfing KJ, Leonardi G, Nomikou I, Raczaszek-Leonardi J, Hullermeier E. Multimodal turn-taking: Motivations, methodological challenges, and novel approaches. IEEE Trans Cogn Dev Syst. 2020;12: 260–271. doi: 10.1109/TCDS.2019.2892991

[7] Brône G, Oben B, Jehoul A, Vranjes J, Feyaerts K. Eye gaze and viewpoint in multimodal interaction management. Cogn Linguist. 2017;28: 449–483. doi: 10.1515/cog-2016-0119

[8] Richardson MJ, Chemero A. Complex Dynamical Systems and Embodiment Self-organization of cognitive processes. In: editor L. S,. The Routledge handbook of embodied cognition. Routledge; 2014. pp. 39–50. Available: https://www.researchgate.net/publication/321176809

[9] daSilva EB, Wood A. How and Why People Synchronize: An Integrated Perspective. Personal Soc Psychol Rev. 2024; 1–29. doi: 10.1177/10888683241252036 38770754

[10] Garrod S, Pickering MJ. Why is conversation so easy? Trends Cogn Sci. 2004;8: 8–11. doi: 10.1016/j.tics.2003.10.016 14697397

[11] Miles K, Weisser A, Kallen RW, Varlet M, Richardson MJ, Buchholz JM. Behavioral dynamics of conversation, (mis)communication and coordination in noisy environments. Sci Rep. 2023;13: 20271. doi: 10.1038/s41598-023-47396-y 37985887 PMC10662155

[12] Shockley K, Richardson DC, Dale R. Conversation and Coordinative Structures. Top Cogn Sci. 2009;1: 305–319. doi: 10.1111/j.1756-8765.2009.01021.x 25164935

[13] Coco MI, Dale R, Keller F. Performance in a Collaborative Search Task: The Role of Feedback and Alignment. Top Cogn Sci. 2018;10: 55–79. doi: 10.1111/tops.12300 29131516

[14] Dale R, Fusaroli R, Duran ND, Richardson DC. The Self-Organization of human interaction. Psychology of Learning and Motivation—Advances in Research and Theory. 2013. pp. 43–95. doi: 10.1016/B978-0-12-407187-2.00002–2

[15] Louwerse MM, Dale R, Bard EG, Jeuniaux P. Behavior matching in multimodal communication Is synchronized. Cogn Sci. 2012;36: 1404–1426. doi: 10.1111/j.1551-6709.2012.01269.x 22984793

[16] Fusaroli R, Tylén K. Investigating Conversational Dynamics: Interactive Alignment, Interpersonal Synergy, and Collective Task Performance. Cogn Sci. 2016;40: 145–171. doi: 10.1111/cogs.12251 25988263

[17] Dale R, Kirkham NZ, Richardson DC. The Dynamics of Reference and Shared Visual Attention. Front Psychol. 2011;2: 1–11. doi: 10.3389/FPSYG.2011.00355 22164151 PMC3230789

[18] Richardson DC, Dale R. Looking to understand: The coupling between speakers’ and listeners’ eye movements and its relationship to discourse comprehension. Cogn Sci. 2005;29: 1045–1060. doi: 10.1207/s15516709cog0000_29 21702802

[19] Richardson DC, Dale R, Kirkham NZ. The art of conversation is coordination: Common ground and the coupling of eye movements during dialogue: Research article. Psychol Sci. 2007;18: 407–413. doi: 10.1111/j.1467-9280.2007.01914.x 17576280

[20] Bischof WF, Anderson NC, Kingstone A. Temporal Methods for Eye Movement Analysis. In: Klein C, Ettinger U, editors. Eye Movement Research Studies in Neuroscience, Psychology and Behavioral Economics. Springer; 2019. pp. 407–448. doi: 10.1007/978-3-030-20085-5_10

[21] Gobel MS, Kim HS, Richardson DC. The dual function of social gaze. Cognition. 2015;136: 359–364. doi: 10.1016/j.cognition.2014.11.040 25540833

[22] Liszkowski U, Carpenter M, Henning A, Striano T, Tomasello M. Twelve-month-olds point to share attention and interest. Dev Sci. 2004;7: 297–307. doi: 10.1111/j.1467-7687.2004.00349.x 15595371

[23] Tomasello M. If They’re So Good at Grammar, Then Why Don’t They Talk? Hints From Apes’ and Humans’ Use of Gestures. Lang Learn Dev. 2007;3: 133–156. doi: 10.1080/15475440701225451

[24] Krause MA, Fouts RS. Chimpanzee (Pan troglodytes) pointing: Hand shapes, accuracy, and the role of eye gaze. J Comp Psychol. 1997;111: 330–336. doi: 10.1037/0735-7036.111.4.330 9419879

[25] Caruana N, Inkley C, Nalepka P, Kaplan DM, Richardson MJ. Gaze facilitates responsivity during hand coordinated joint attention. Sci Rep. 2021;11: 21037. doi: 10.1038/s41598-021-00476-3 34702900 PMC8548595

[26] Bloch C, Tepest R, Jording M, Vogeley K, Falter-Wagner CM. Intrapersonal synchrony analysis reveals a weaker temporal coherence between gaze and gestures in adults with autism spectrum disorder. Sci Rep. 2022;12: 20417. doi: 10.1038/s41598-022-24605-8 36437262 PMC9701674

[27] Langton SRH, Bruce V. You must see the point: Automatic processing of cues to the direction of social attention. J Exp Psychol Hum Percept Perform. 2000;26: 747–757. doi: 10.1037//0096-1523.26.2.747 10811173

[28] Goodwin C. Gestures as a resource for the organization of mutual orientation. 1986;62: 29–50. doi: 10.1515/semi.1986.62.1–2.29

[29] Pagnotta M, Laland KN, Coco MI. Attentional coordination in demonstrator-observer dyads facilitates learning and predicts performance in a novel manual task. Cognition. 2020;201: 104314. doi: 10.1016/j.cognition.2020.104314 32454263

[30] Yu C, Smith LB. Joint attention without gaze following: Human infants and their parents coordinate visual attention to objects through eye-hand coordination. PLoS ONE. 2013;8: e79659. doi: 10.1371/journal.pone.0079659 24236151 PMC3827436

[31] Yu C, Smith LB. Hand–Eye Coordination Predicts Joint Attention. Child Dev. 2017;88: 2060–2078. doi: 10.1111/cdev.12730 28186339 PMC6894731

[32] Vesper C, Richardson MJ. Strategic communication and behavioral coupling in asymmetric joint action. Exp Brain Res. 2014;232: 2945–2956. doi: 10.1007/s00221-014-3982-1 24838557 PMC4381276

[33] Henrich J, Gil-White FJ. The evolution of prestige Freely conferred deference as a mechanism for enhancing the benefits of cultural transmission. Evol Hum Behav. 2001;22: 165–196. doi: 10.1016/s1090-5138(00)00071-4 11384884

[34] Redhead D, Cheng JT, Driver C, Foulsham T, O’Gorman R. On the dynamics of social hierarchy: A longitudinal investigation of the rise and fall of prestige, dominance, and social rank in naturalistic task groups. Evol Hum Behav. 2019;40: 222–234. doi: 10.1016/j.evolhumbehav.2018.12.001

[35] Van Vugt M, Hogan R, Kaiser RB. Leadership, Followership, and Evolution: Some Lessons From the Past. Am Psychol. 2008;63: 182–196. doi: 10.1037/0003-066X.63.3.182 18377108

[36] Nikoleizig L, Schmukle SC, Griebenow M, Krause S. Investigating contributors to performance evaluations in small groups: Task competence, speaking time, physical expressiveness, and likability. PLoS ONE. 2021;16: e0252980. doi: 10.1371/journal.pone.0252980 34111193 PMC8191988

[37] Foulsham T, Cheng JT, Tracy JL, Henrich J, Kingstone A. Gaze allocation in a dynamic situation: Effects of social status and speaking. Cognition. 2010;117: 319–331. doi: 10.1016/j.cognition.2010.09.003 20965502

[38] Thomas-Hunt MC, Phillips KW. When What You Know Is Not Enough: Expertise and Gender Dynamics in Task Groups. Pers Soc Psychol Bull. 2004;30: 1585–1598. doi: 10.1177/0146167204271186 15536241

[39] Ellyson SL, Dovidio JF, Fehr BJ. Visual Behavior and Dominance in Women and Men. Gender and Nonverbal Behavior. New York, NY: Springer New York; 1981. pp. 63–79. doi: 10.1007/978-1-4612-5953-4_4

[40] Hall J, Watson WH. The Effects of a Normative Intervention on Group Decision-Making Performance. Hum Relat. 1970;23: 299–317. doi: 10.1177/001872677002300404

[41] Onkhar V, Dodou D, de Winter JCF. Evaluating the Tobii Pro Glasses 2 and 3 in static and dynamic conditions. Behav Res Methods. 2023;1: 1–18. doi: 10.3758/s13428-023-02173-7 37550466 PMC11289326

[42] Nasiopoulos E, Risko EF, Foulsham T, Kingstone A. Wearable computing: Will it make people prosocial? Br J Psychol. 2015;106: 209–216. doi: 10.1111/bjop.12080 25040108

[43] Hessels RS, Benjamins JS, Niehorster DC, van Doorn AJ, Koenderink JJ, Holleman GA, et al. Eye contact avoidance in crowds: A large wearable eye-tracking study. Atten Percept Psychophys. 2022;84: 2623–2640. doi: 10.3758/s13414-022-02541-z 35996058 PMC9630249

[44] Olsen A. The Tobii I-VT Fixation Filter: Algorithm description. Tobii Technol. 2012 p. 21.

[45] Bakeman R, Quera V. Sequential analysis and observational methods for the behavioral sciences. Sequential Analysis and Observational Methods for the Behavioral Sciences. Cambridge University Press; 2011. doi: 10.1017/CBO9781139017343

[46] Mangold. INTERACT Benutzerhandbuch. Mangold International GmbH; 2020. Available: www.mangold-international.com

[47] Rogers SL, Speelman CP, Guidetti O, Longmuir M. Using dual eye tracking to uncover personal gaze patterns during social interaction. Sci Rep. 2018;8: 4271. doi: 10.1038/s41598-018-22726-7 29523822 PMC5844880

[48] Jording M, Hartz A, Bente G, Schulte-Rüther M, Vogeley K. The “Social Gaze Space”: A Taxonomy for Gaze-Based Communication in Triadic Interactions. Front Psychol. 2018;9: 226. doi: 10.3389/fpsyg.2018.00226 29535666 PMC5834481

[49] Cicchetti DV. Guidelines, Criteria, and Rules of Thumb for Evaluating Normed and Standardized Assessment Instruments in Psychology. Psychol Assess. 1994;6: 284–290. doi: 10.1037/1040-3590.6.4.284

[50] Marwan N, Carmen Romano M, Thiel M, Kurths J. Recurrence plots for the analysis of complex systems. Phys Rep. 2007;438: 237–329. doi: 10.1016/j.physrep.2006.11.001

[51] Fusaroli R, Konvalinka I, Wallot S. Analyzing Social Interactions: The promises and challenges of using Cross Recurrence Quantification Analysis. From mathematical theory to real-world applications. Springer Proceedings in Mathematics & Statistics. Springer International Publishing; 2014. pp. 137–155.

[52] Dale R, Warlaumont AS, Richardson DC. Nominal cross recurrence as a generalized lag sequential analysis for behavioral streams. Int J Bifurc Chaos. 2011;21: 1153–1161. doi: 10.1142/S0218127411028970

[53] Anderson NC, Bischof WF, Laidlaw KEW, Risko EF, Kingstone A. Recurrence quantification analysis of eye movements. Behav Res Methods. 2013;45: 842–856. doi: 10.3758/s13428-012-0299-5 23344735

[54] Marwan N, Kurths J. Nonlinear analysis of bivariate data with cross recurrence plots. Phys Lett Sect Gen At Solid State Phys. 2002;302: 299–307. doi: 10.1016/S0375-9601(02)01170-2

[55] Coco MI, Dale R. Cross-recurrence quantification analysis of categorical and continuous time series: an R package. Front Psychol. 2014;5: 1–14. doi: 10.3389/FPSYG.2014.00510 25018736 PMC4073592

[56] Wallot S, Leonardi G. Analyzing multivariate dynamics using cross-recurrence quantification analysis (CRQA), diagonal-cross-recurrence profiles (DCRP), and multidimensional recurrence quantification analysis (MdRQA)—A tutorial in R. Front Psychol. 2018;9: 2232. doi: 10.3389/fpsyg.2018.02232 30564161 PMC6288366

[57] TheMathWorksInc. MATLAB version: 9.14.0 (R2023a). Natick, Massachusetts, United States: The MathWorks Inc.; 2023. Available: https://www.mathworks.com

[58] Coco MI, Mønster D, Leonardi G, Dale R, Wallot S. Unidimensional and Multidimensional Methods for Recurrence Quantification Analysis with crqa. R J. 2021;13: 145–163. doi: 10.32614/rj-2021-062

[59] Amrhein V, Greenland S, McShane B. Scientists rise up against statistical significance. Nature. 2019;567: 305–307. doi: 10.1038/d41586-019-00857-9 30894741

[60] Malcolm GL, Shomstein S. Object-based attention in real-world scenes. J Exp Psychol Gen. 2015;144: 257–263. doi: 10.1037/xge0000060 25844622

[61] Theeuwes J, Mathôt S, Kingstone A. Object-based eye movements: The eyes prefer to stay within the same object. Atten Percept Psychophys. 2010;72: 597–601. doi: 10.3758/APP.72.3.597 20348565

[62] Lu Z, van Zoest W. Combining social cues in attention: Looking at gaze, head, and pointing cues. Atten Percept Psychophys. 2023;85: 1021–1033. doi: 10.3758/s13414-023-02669-6 36849577 PMC10167180

