# Supplementary material for: Looking, pointing, and talking together: How dyads of differential expertise coordinate attention during conversation
Source: PLoS One. 2024 Dec 19;19(12):e0315728. doi: 10.1371/journal.pone.0315728 (PMC11658607; doi:10.1371/journal.pone.0315728)
Supplement: S1 Appendix — (PDF) [file pone.0315728.s001.pdf]

S1 Appendix. Training & Self-Rated Expertise Questionnaire Items

Training

Participants were randomly assigned to one of two classrooms and received a 30-minute training on animal biology. They were informed that the training content would be relevant for a later task. Both groups were shown the same set of slides on wildlife animals; however, the information verbally presented differed between the two groups. One group received information relevant to visually estimating the age of animals, while the other group was given trivia unrelated to age estimation.

In the training designed to improve age estimation skills, participants learned about the age ranges of different animals, which body features are reliable indicators of age, and which are not. For example, they were informed that in contrast to animals' posture, neither apparent pelt thickness nor body size is a good age indicator, as these features can vary with the season or the animal's gender. Further, some trivia about the animals was mentioned, such as animals' roaming and mating behaviors, evolutionary origins, diets, and habitats. In contrast, the trivia-only training solely and more extensively covered those facts unrelated to age estimation.

Self-Rated Expertise Questionnaire Items

To assess participants' A) self-rating of their own performance on the individual task (before the conversation) as well as their B) perceived expertise relative to their conversation partner (after the conversation), we created and used the following German questionnaire items (English translation in brackets).

A) Self-rating of own performance in individual task

Participants rated their agreement with each statement on a 6-point Likert scale, where 1 indicated "Trifft Überhaupt nicht zu" (Does not apply at all), and 6 indicated "Trifft vollkommen zu" (Fully applies). The measure was calculated as the average score across the items listed below:

- "Ich habe die Aufgabe, das Alter der Tiere richtig einzuschätzen, insgesamt gut gelöst." (I solved the task of estimating the animals' ages well overall.)
- "Ich habe das Alter der Tiere meistens korrekt eingeschätzt." (I mostly estimated the age of the animals correctly.)
- "Ich war mir mit den Einschätzungen vom Alter meistens sicher." (I was mostly confident in my age estimations.)

B) Self-Rating of Relative Expertise

Participants rated each item on a 7-point scale, with 1 indicating "Die andere Person" (The other person), 4 indicating "Beide gleich" (Both equally), and 7 indicating "Ich" (Myself). The measure was calculated as the average score across these items:

- "Wer war im Allgemeinen eher der Experte / die Expertin in dieser Angelegenheit?" (Who was generally more of an expert in this matter?)
- "Wer hat die besten Informationen beigetragen, durch die Ihr Entscheidungen treffen konntet?" (Who contributed the best information for making decisions?)
- "Wer war kompetenter in dieser Angelegenheit?" (Who was more competent in this matter?)
