# Supplementary material for: Looking, pointing, and talking together: How dyads of differential expertise coordinate attention during conversation
Source: PLoS One. 2024 Dec 19;19(12):e0315728. doi: 10.1371/journal.pone.0315728 (PMC11658607; doi:10.1371/journal.pone.0315728)
Supplement: S2 Appendix — (PDF) [file pone.0315728.s002.pdf]

S2 Appendix. Behavioral Coding Rules

The coding scheme was created in line with recommendations from Bakeman and Quera (2011). All behaviors were coded in a mutually exclusive and exhaustive manner. This means regarding turn taking it was “always someone’s” turn and “never no-one’s” turn. Regarding gaze behavior, there was always “something that was looked at”. If gaze was directed outside the predefined areas of interest (the items) this was coded with the label “none”. Regarding pointing, the same is true, only that any time the participant was not pointing is coded as “nopoint”. Before performing cross recurrence quantification analysis, for each participant those non-relevant codes “none” and “nopoint” were recoded to be unique for this participant. This excludes them from recurring with their respective interlocutor’s non-relevant codes (Otherwise, two people’s gazes outside the items or their absence of pointing would count as “looking / pointing at the same thing”).

Listed below are the behavior-specific rules used by the coders:

Gaze

- Gaze onto a picture was defined as the time between the first frame when the gaze cursor was visible within the picture until the last frame where it was visible within that same picture.
- When a participant looked at a picture and the gaze cursor disappeared for shorter than 1 second and reappeared inside the same area of interest (e.g. when pupils could not be tracked continuously due to blinks or quick head movements like nodding or head shaking), the preceding code defined the code for the whole time the gaze cursor was missing.

Pointing

- A "point" event began with the first frame where a part of the hand or finger was seen in a forward movement that clearly ended in a deictic gesture. The same “point" event ended with the first frame in which a retraction of the hand or finger was visible.
- When there was a transition of “pointing” from one picture directly to another one, the change in codes occurred as soon as the hand movement towards the subsequent picture began.
- Some gestures were not easily identified as "deictic" without taking the accompanying verbal reference to what was meant into account. (e.g. someone waving their hand in a vague direction could also be a result of them trying make a point of an argument without an indication of direction). In these cases, audio was checked. Only if it was clear that the directed hand / finger was used to reference something (e.g. the speaker uses verbal deictic references such as “this”, or “the upper one here”), it was coded as “pointing”. Else, the hand movement was disregarded.

Turn-taking

- Turn talking was coded in an “exhaustive” manner, meaning there was “always someone’s” turn and “never no-one’s” turn. In moments of silence, the preceding code continued until the interlocutor started their turn with the first audible utterance.
- The conversation began as soon as the door to the room was closed by the experimenter leaving the room. Participants were told to start the conversation at this mark. The turn-code of the first participant to start speaking extended to the start of the recording, even if there was a brief moment of silence in the beginning. The conversation ended with the end of the last turn-code of the participant that was speaking before the experimenter entered the room again.
- Verbal backchannels such as “ehm”, “uhm”, “mhm” and “ok” were not considered as turns as these could not be defined in a meaningful way, as (1) these utterances are short and occur often and (2) most of these utterances happen without the other speaker stopping to talk
- In the same manner, unsuccessful interruptions (i.e., a listener trying to start talking but the speaker does not stop to talk) were not coded as turns. With successful interruptions at which there was a moment where both participants were speaking at the same time, the switch in turns was coded as soon as the interrupter was the only one heard speaking.
