# Supplementary material for: Looking, pointing, and talking together: How dyads of differential expertise coordinate attention during conversation
Source: PLoS One. 2024 Dec 19;19(12):e0315728. doi: 10.1371/journal.pone.0315728 (PMC11658607; doi:10.1371/journal.pone.0315728)
Supplement: S3 Appendix — (PDF) [file pone.0315728.s003.pdf]

S3 Appendix. Cross Recurrence

This appendix defines cross-recurrence, cross-recurrence modified by temporal parameters (e.g., by which of the dialogue partners is speaking), the diagonal cross-recurrence profile DCRP, and the cross-recurrence CORM.

Consider two event sequences $\boldsymbol{E} = e_i$, i=1, …, N and $\boldsymbol{F} = f_j$, j=1, …, N. The events could consist, for example, of gaze fixations or pointing actions. Two events e_i and f_j are cross-recurrent if they belong to the same category, for example, two fixations of the same object or pointing to the same object. A cross-recurrence matrix R with entries r_{ij} is defined as follows:

$$r_{\mathrm{ij}} = \begin{cases} 1, & \mathrm{e}_i \text{ and } \mathrm{f}_j \text{ are cross–recurrent} \\ 0, & \text{otherwise} \end{cases}.$$

We define the *recurrence rate* (RR) as the percentage of recurrent entries,

$$\mathrm{RR} = \frac{100}{N^2} \sum_{i=1}^{N} \sum_{j=1}^{N} r_{ij}$$

On the basis of the recurrence matrix, we define the *diagonal cross recurrence profile* (DCRP), which represents the recurrence along lines parallel to the main diagonal. Given w with w < N,

$$\mathrm{DCRP}(w) = \begin{cases} \dfrac{100}{N - w} \sum_{k=1}^{N-w} r_{k+w,k} & \text{if } w \geq 0 \\ \dfrac{100}{N + w} \sum_{k=1}^{N+w} r_{k,k-w} & \text{if } w < 0 \end{cases}$$

The *center of recurrence mass* (CORM) is defined as the distance of the center of gravity of all recurrences from the main diagonal, normalized such that the possible values are in the range [-100, 100] (see [20]).

$$\mathrm{CORM} = \frac{100}{(N-1)C} \sum_{i=1}^{N} \sum_{j=1}^{N} (j - i) r_{ij},$$

where $\mathrm{C} = \sum_{\mathrm{i}=1}^{\mathrm{N}} \sum_{\mathrm{j}=1}^{\mathrm{N}} \mathrm{r}_{\mathrm{ij}}$. The CORM measure indicates the dominant lag of cross-recurrences. Small absolute corm values indicate that same-category events in both sequences tend to occur close in time, whereas large absolute corm values indicate that the cross-recurrences tend to

occur with either a large positive or negative lag. CORM values are positive if the sequence ***E*** is leading the sequence ***F***, and it is negative if the sequence ***E*** is trailing the sequence ***F***.

Note that the cross-recurrence CORM defined above differs from the auto-recurrence CORM defined by [51]. In that case, CORM was defined for an auto-recurrence matrix of a sequence of fixations with itself. Given the symmetry of the auto-recurrence matrix, CORM was computed for only half the recurrence matrix, with possible values in the range [0, 100].

The cross-recurrence matrix defined above can be modified by temporal parameters, for example whether the trained or the untrained participant is speaking. This is illustrated with example data (see Table S3.1.) in Figure S3.1. Panel A shows a simple cross-recurrence matrix over 10 time points. Now assume that the trained participant is speaking at time points 1, 2, 3, 6, 7, and 8, and the untrained participant is speaking at time points 4, 5, 9, and 10. Panel B shows the cross-recurrence matrix of panel A with recurrences involving only time points when the trained participant is speaking. This is illustrated by the bar below the recurrence matrix. All recurrences involving other time points are eliminated. Panel C shows the cross-recurrence matrix of panel A with recurrences involving only time points when the untrained participant is speaking, again as illustrated by the bar below the recurrence matrix. All recurrences involving other time points are eliminated. The measures DCRP and CORM that depend on the speaking turn, i.e. whether the trained or the untrained participant is speaking, are based on the cross-recurrence matrices in panels B and C.

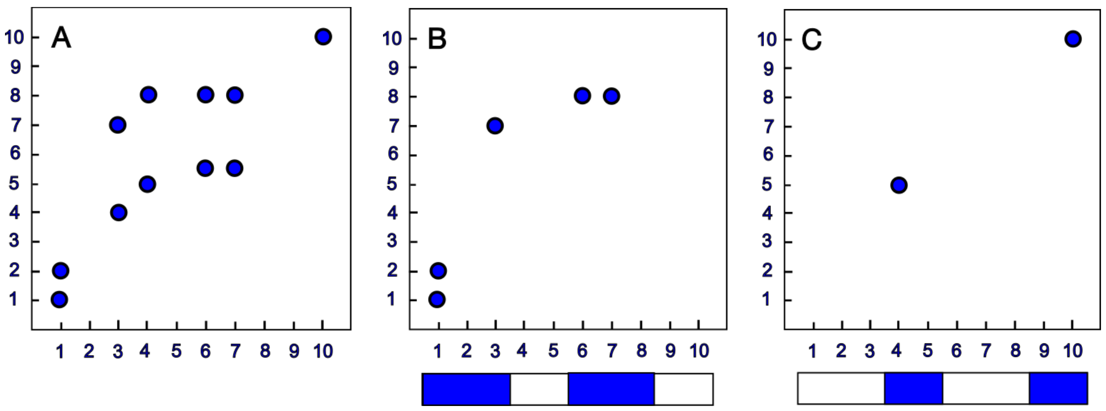

Figure S3.1.

A) shows a simple cross-recurrence matrix over 10 time points, with recurrences indicated by filled circles. B) shows the cross-recurrence matrix of panel A with recurrences involving only time points when the trained participant is speaking, in this example time points 1, 2, 3, 6, 7, and 8. This is illustrated by the bar below the recurrence matrix. All recurrences involving other time points are eliminated. C) shows the cross-recurrence matrix of panel A with recurrences involving only time points when the untrained participant is speaking, i.e., time points 4, 5, 9 10, again illustrated by the bar below the recurrence matrix. All recurrences involving other time points are eliminated.

time point	trained gaze	untrained gaze	turn	trained gaze while trained turn	untrained gaze while trained turn	trained gaze while untrained turn	untrained gaze while untrained turn
1	A	A	tr	A	A		
2	-	A	tr		A		
3	B	-	tr	B			
4	C	B	utr			C	B
5	-	C	utr				C
6	C	-	tr	C			
7	C	B	tr	C	B		
8	-	C	tr		C		
9	-	-	utr				
10	D	D	utr			D	D

Table S3.1

Example time series data from which recurrence plots seen in Figure 3.1 were constructed. The first 4 columns ‘time point’, ‘trained gaze’, ‘untrained gaze’ and ‘turn’ represent time series data of two participants’ (one trained, one untrained) gaze towards areas of interest A, B, and so on as well was information on who was speaking at each given time point. Figure S3.1, panel A shows the recurrence plot relating to ‘trained gaze’ and ‘untrained gaze’ columns. The last 4 columns ‘… gaze while … turn’ represent respective gaze data conditional on turns. Figure S3.1, panels B and C show respective recurrence plots of trained and untrained individuals’ gaze while only one (either trained or untrained) is speaking.
