# Supplementary material for: Looking, pointing, and talking together: How dyads of differential expertise coordinate attention during conversation
Source: PLoS One. 2024 Dec 19;19(12):e0315728. doi: 10.1371/journal.pone.0315728 (PMC11658607; doi:10.1371/journal.pone.0315728)
Supplement: S4 Appendix — (PDF) [file pone.0315728.s004.pdf]

S4 Appendix. All DCRPs and ANOVA results

In the Results section we reported that all mentioned DCRPs were found to be coupled at above chance levels. Below we report all respective DCRPs together with each a 2 (profiles; coupled behaviors vs. randomized baseline) × 43 (lags) mixed-design analysis of variance (ANOVA; with lag as the repeated measures factor). Each comparison of a profile to its randomized baseline shows the same pattern of a significant main effect for both profile and lag as well as an interaction of both. This means that all DCRPs under investigation were coupled at above chance levels and to varying degrees across lags.

Measure	Effect	*df*	*F*
1) tr_gaze_utr_gaze | tr_speaking	Lags	42	74.93***
	Profiles	1	1478.29***
	Lags:Profiles	42	7.01***
2) tr_gaze_utr_gaze | utr_speaking	Lags	42	39.14***
	Profiles	1	1075.84***
	Lags:Profiles	42	11.23***
3) tr_point_tr_gaze	Lags	42	15.66***
	Profiles	1	636.86***
	Lags:Profiles	42	2.98***
4) utr_point_utr_gaze	Lags	42	8.86***
	Profiles	1	575.33***
	Lags:Profiles	42	2.39***
5) tr_point_utr_gaze	Lags	42	12.52***
	Profiles	1	677.47***
	Lags:Profiles	42	2.64***
6) utr_point_tr_gaze	Lags	42	8.86***
	Profiles	1	575.33***
	Lags:Profiles	42	2.39***

Note. tr = trained, utr = untrained. *** $p < .001$

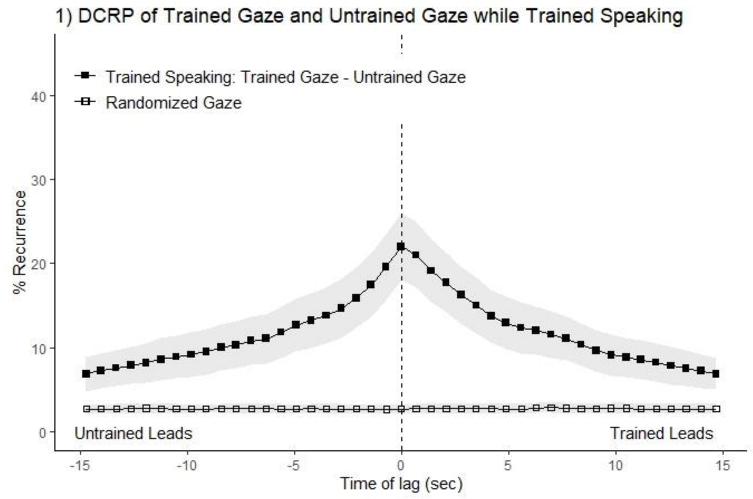
1) DCRP of Trained Gaze and Untrained Gaze while Trained Speaking
Trained Speaking: Trained Gaze - Untrained Gaze
Randomized Gaze
% Recurrence
Untrained Leads
Trained Leads
Time of lag (sec)

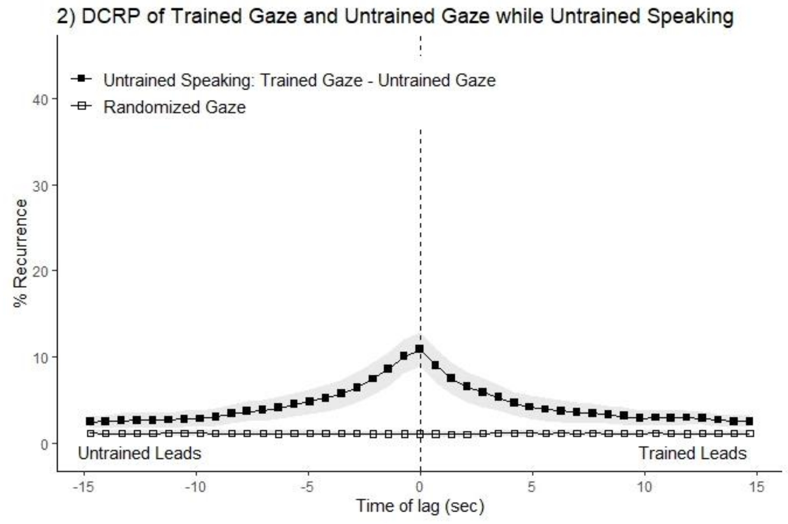
2) DCRP of Trained Gaze and Untrained Gaze while Untrained Speaking
Untrained Speaking: Trained Gaze - Untrained Gaze
Randomized Gaze
% Recurrence
Untrained Leads
Trained Leads
Time of lag (sec)

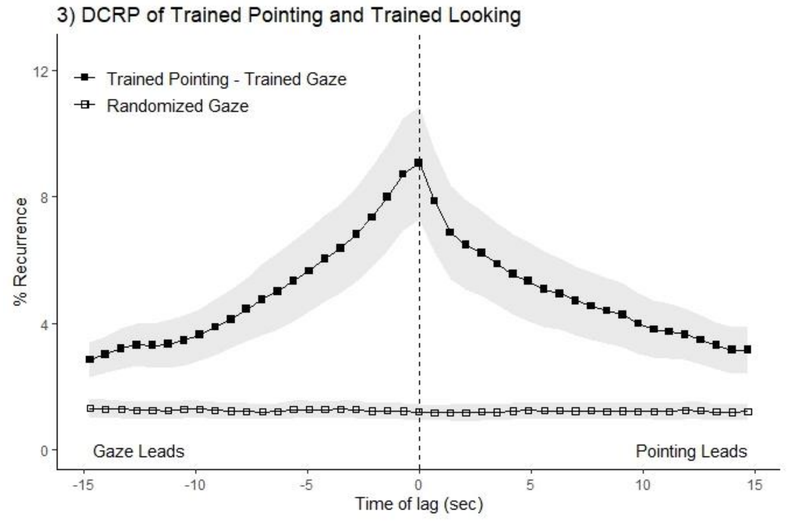
3) DCRP of Trained Pointing and Trained Looking
Trained Pointing - Trained Gaze
Randomized Gaze
% Recurrence
Gaze Leads
Pointing Leads
Time of lag (sec)

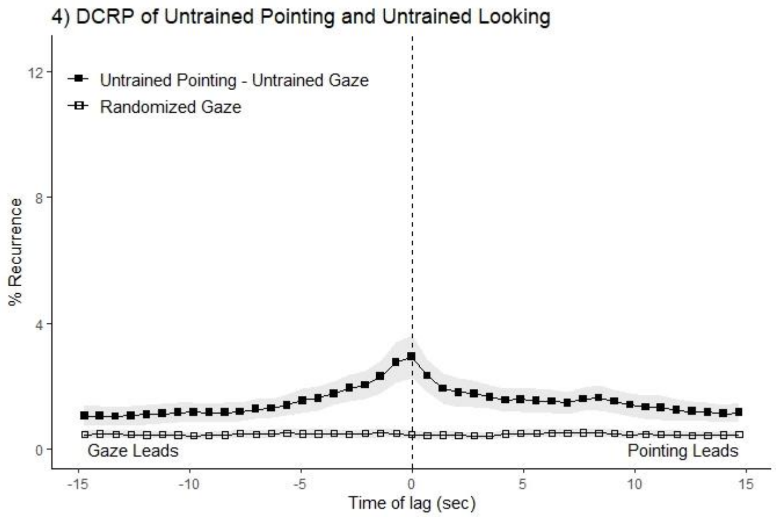
4) DCRP of Untrained Pointing and Untrained Looking
Untrained Pointing - Untrained Gaze
Randomized Gaze
% Recurrence
Gaze Leads
Pointing Leads
Time of lag (sec)

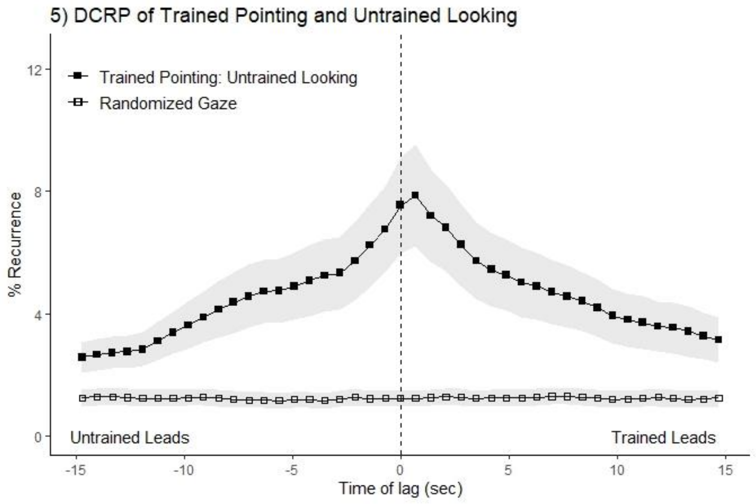
5) DCRP of Trained Pointing and Untrained Looking
Trained Pointing: Untrained Looking
Randomized Gaze
% Recurrence
Untrained Leads
Trained Leads
Time of lag (sec)

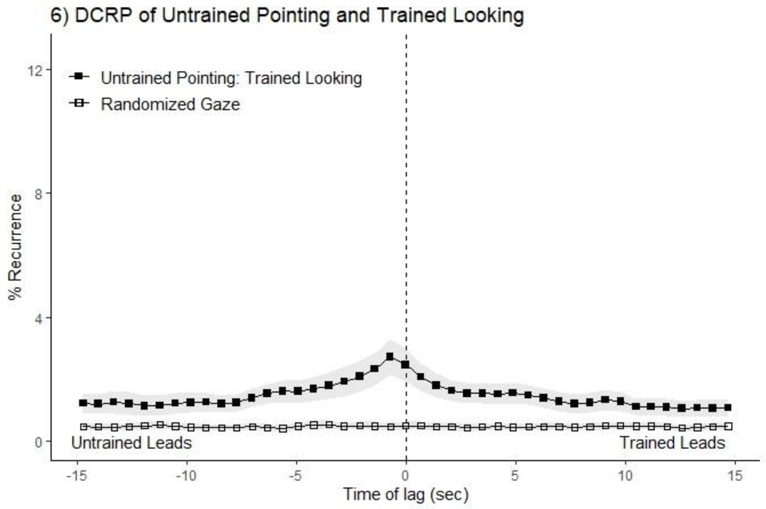
6) DCRP of Untrained Pointing and Trained Looking
Untrained Pointing: Trained Looking
Randomized Gaze
% Recurrence
Untrained Leads
Trained Leads
Time of lag (sec)
